# Simplifying Macroinvertebrate Biodiversity Assessment: A Standardised Metabarcoding Method for Invertebrate Samples With High Volumes of Debris

**DOI:** 10.1111/1755-0998.70213

**Published:** 2026-10-04

**Authors:** Melissa Carew, Jessica Chung, Geneveive Hehir, Ary Hoffmann, Rhys Coleman

**Affiliations:** ^1^ Waterway Ecosystem Research Group (WERG), School of Ecosystem and Forest Sciences The University of Melbourne Richmond Victoria Australia; ^2^ Pest and Environmental Adaptation Research Group (PEARG), School of BioSciences, Bio21 Institute The University of Melbourne Melbourne Victoria Australia; ^3^ Melbourne Bioinformatics The University of Melbourne Melbourne Victoria Australia; ^4^ Research and Modelling Melbourne Water Docklands Victoria Australia

**Keywords:** biodiversity, bioinformatics, invertebrates, macroinvertebrates, metabarcoding, workflow

## Abstract

DNA metabarcoding of freshwater macroinvertebrate samples can provide rapid and accurate identification of species diversity. Processing samples where macroinvertebrates are not separated from net contents can substantially reduce time and cost. We devised and tested a standardised method for processing combined kick/sweep samples using DNA metabarcoding to provide accurate estimates of macroinvertebrate species diversity. Kick and sweep samples were collected from 10 sites across Greater Melbourne known to vary in catchment land use, macroinvertebrate abundance and diversity as well as the type and volume of sampling debris. A sieving, blending and DNA extraction protocol was developed to reduce large debris, rocks and sand in samples, and to concentrate macroinvertebrates for metabarcoding. Variable percentages of the dry weight of samples from each site were used as a source of DNA for metabarcoding. We found that the macroinvertebrate species diversity recovered was mostly consistent when processing different percentages (20%–40%) of dry weight, but a minimum dry weight would be recommended for samples with low total dry weight. We present a method with a laboratory and bioinformatic workflow that enables the cost‐effective and reliable processing of large volume samples, like those encountered in freshwater bioassessment. This metabarcoding approach has potential to be applied to any invertebrate biodiversity analysis where invertebrates are not separated from sampling debris, regardless of volume and composition, and used on samples varying in species abundance, diversity and density.

## 1 Introduction

DNA metabarcoding has been successfully used to provide species‐level identification of freshwater macroinvertebrates for biodiversity assessment (Carew et al. 2018; Elbrecht et al. 2019; Hajibabaei et al. 2011). DNA metabarcoding methods employed for assessing freshwater biodiversity can be broadly categorised as sorted ‘tissue‐based’ methods (e.g., Carew et al. 2013; Hajibabaei et al. 2011), unsorted ‘bulk’ methods (e.g., Carew et al. 2021; Majaneva et al. 2018) and ‘environmental DNA (eDNA)’ methods (e.g., Aylagas et al. 2016; Beentjes et al. 2017). Like traditional morphological methods, tissue‐based methods use net‐collected macroinvertebrates that have been sorted from debris prior to DNA metabarcoding. Bulk methods also use net‐collected macroinvertebrates, but sampling debris along with macroinvertebrates are processed with DNA metabarcoding. Environmental DNA metabarcoding relies on DNA sourced from filtered water or sediment samples.

Typically, the macroinvertebrate composition and diversity revealed by tissue‐based DNA metabarcoding strongly overlap with traditional morphological approaches (Carew et al. 2018; Elbrecht, Vamos, et al. 2017). Similar patterns of macroinvertebrate composition and diversity have also been observed using bulk samples (Carew et al. 2021; Pereira‐da‐Conceicoa et al. 2020). However, using current approaches, there is typically little overlap between macroinvertebrate species composition and biodiversity measures obtained from eDNA metabarcoding and those from traditionally sampled (net‐collected) material (Hajibabaei et al. 2019; Macher and Leese 2017). While eDNA samples can reduce sample collection time and remove the need for sorting, net‐collected samples tend to detect greater in situ macroinvertebrate biodiversity (Gleason et al. 2021; Hajibabaei et al. 2019; Pereira‐da‐Conceicoa et al. 2020).

Costs involved in DNA metabarcoding of net‐collected macroinvertebrates have already been shown to be lower compared to traditional methods and can be reduced further if samples are not sorted prior to DNA metabarcoding (Buchner et al. 2021; Carew et al. 2021; Majaneva et al. 2018; Nichols et al. 2019; Pereira‐da‐Conceicoa et al. 2020). While sorting allows for non‐target taxa and debris to be removed, and adjusts for differences in taxon relative abundance (e.g., Chessman 1995; Resh et al. 1995; Walsh 1997), the time, effort and expertise required can be substantial (Carew et al. 2021). To circumvent this, strategies have been trialled for using bulk material. Majaneva et al. (2018) found that plant‐based DNA extraction kits were suited for extracting DNA from unsorted kick samples when taking small sub‐samples of the blended homogenate. However, they suggested that samples with low abundance would likely provide more stable results if specimens were sorted prior to metabarcoding. Nichols et al. (2019) reliably detected macroinvertebrates from unsorted kick samples, even if animals were removed prior to DNA metabarcoding, as residual DNA/animals remained. Pereira‐da‐Conceicoa et al. (2020) found unsorted macroinvertebrate samples yielded higher macroinvertebrate biodiversity compared to tissue‐based methods. To increase the efficiency of processing unsorted samples, Buchner et al. (2021) developed a sample blending protocol which was able to recover 80% of taxonomic units per sample replicate, while Carew et al. (2021) used a seiving method to reduce the volume of sweep samples for metabarcoding and detected similar macroinvertebrate biodiversity compared to sorted samples. Overall, these studies show that homogenising and metabarcoding of bulk material can provide accurate estimates of aquatic macroinvertebrate biodiversity. Therefore, this raises the issue of whether an approach that uses combined samples, often with very high volumes of material, that incorporates all habitats traditionally sampled for standard macroinvertebrate biomonitoring assessments (i.e., Barbour et al. 1999; Chessman 1995; Hering et al. 2004) could be developed.

In this study, we develop and refine a protocol to standardise the processing of unsorted macroinvertebrates from combined kick and sweep samples for DNA metabarcoding. Although bulk metabarcoding approaches are increasingly used, the volume of unsorted material required to reliably assess biodiversity from large, debris‐rich samples has not been systematically evaluated. In combined kick/sweep samples, macroinvertebrate diversity and abundance can vary substantially, as can the quantity and type of sampling debris, potentially influencing species detection and comparability among sites. To address this, we build on the sieving‐based approach developed in Carew et al. (2021) to remove large debris, reduce overall sample volume, and concentrate macroinvertebrates relative to debris prior to homogenisation. Macroinvertebrates from sweep and kick samples at each site are combined, sieved, homogenised using a blender, and dried to remove residual ethanol. We then analyse sub‐samples representing 10%, 20%, 30% and 40% of the total dried weight to determine how sample volume influences macroinvertebrate species detection and to establish a standardised, reliable framework for comparing biodiversity across sites. We also present an R‐based workflow for metabarcoding analysis.

## 2 Methods

### 2.1 Site Selection

We selected 10 stream sites across Greater Melbourne, Australia, based on their known abundance and richness of macroinvertebrates from long‐term monitoring by Melbourne Water, the local waterway management authority (https://tools.thewerg.unimelb.edu.au/mwbugs/—Accessed 12 February 2026). These sites were selected to be representative of different sample types that would be found when conducting broad‐scale macroinvertebrate surveys (Table 1). This resulted in four classes of three sites with high abundance and low richness, three sites with low abundance and high richness, two sites with low abundance and low richness, and two sites with high abundance and high richness. Sites were a mixture of urban, forested, and rural and varied in debris composition and volumes.

**TABLE 1:** Site codes and locations across Greater Melbourne, Australia, including the abundance and richness class of macroinvertebrates found at the sites based on long‐term survey data.

Site code	Stream name	Longitude	Latitude	Past collections (1992–2020)				
				Abundance class	Richness class	Site type	Total no. of samples (sweep/kick)	No. of sampling occasions
YAR‐3637‐7	Yarra River	146.0667	−37.7324	High	High	Forest	74	36
FEH‐4184‐3	Fehring Creek	146.0536	−37.7276	High	High	Forest	92	46
FER‐647‐5	Ferny Creek	145.0710	−37.8751	High	Low	Urban	58	27
TOO‐4270‐0	Toomuc Creek	145.4529	−38.0809	High	Low	Rural	22	17
GRD‐7562‐0	Gardiners Creek	145.9433	−37.7218	High	Low	Urban	6	3
DNG‐7957‐4	Dandenong Creek	145.3261	−37.9158	Low	Low	Urban	40	24
DNG‐31881‐3	Dandenong Creek	145.1854	−38.0298	Low	Low	Urban	42	54
BNY‐2904‐2	Bunyip River	145.7464	−37.9343	Low	High	Forest	23	20
UYT‐199‐3	Tributary of Alderman Creek	145.9409	−37.7213	Low	High	Forest	44	23
WM1‐232‐0	Wombat Creek	145.7546	−37.5054	Low	High	Forest	NA^a^	NA^a^

^a^ No previous data but was in the same catchment area as UYT‐199‐3 so was expected to have similar richness and abundance.

### 2.2 Sample Collection

Macroinvertebrates were collected from edge/pool and riffle habitats using standard rapid bioassessment methods (Chessman 1995). In brief, sampling of edge/pool habitats involved sweeping over ~10 m of streambed from wadable areas for about 5–10 min into a 250 μm mesh sweep net. The same approach was used to sample riffle habitats except that a 250 μm mesh kick net was used. All material in sample nets from each habitat was retained and preserved in 1.2 L screw top HDPE 95 mm round jars (Cospak, Australia) with enough absolute ethanol (Chem‐Supply Australia, Australia) added to cover material. Absolute ethanol was replaced 24–48 h after sample collection to ensure the ethanol concentration remained greater than 80%. Samples were then stored at 4°C until further processing.

### 2.3 Sample Processing

Samples from sweep and kick nets were prepared separately for DNA metabarcoding by using three 250 mm entomological sieves, with mesh sizes of 8 mm, 4.75 mm and 250 μm (Figure 1, Supporting Information 1). We kept samples from different habitat types separate at this stage so we could observe if there were any differences in processing. Prior to sieving, samples were placed in a white sorting tray (456 mm × 318 mm × 57 mm) and the composition of samples inspected. The preservation ethanol was decanted using a 250 μm mesh sieve into a clean bucket and retained while the macroinvertebrates and debris were returned to the white sorting tray. For samples containing a high sand content (two kick samples FEH‐418‐3 and YAR‐3637‐7), an extra processing step was added. For these samples, the entire sample was elutriated in the white sorting tray by flushing with high volumes of tap water to suspend the organic content (and most macroinvertebrates), which were decanted into the 250 μm mesh sieve. The sand was then inspected for any remaining macroinvertebrates which were retained. One kick sample with a high sand content (BNY‐2904‐2) was elutriated in the field using a bucket and sampling net.

**FIGURE 1:**
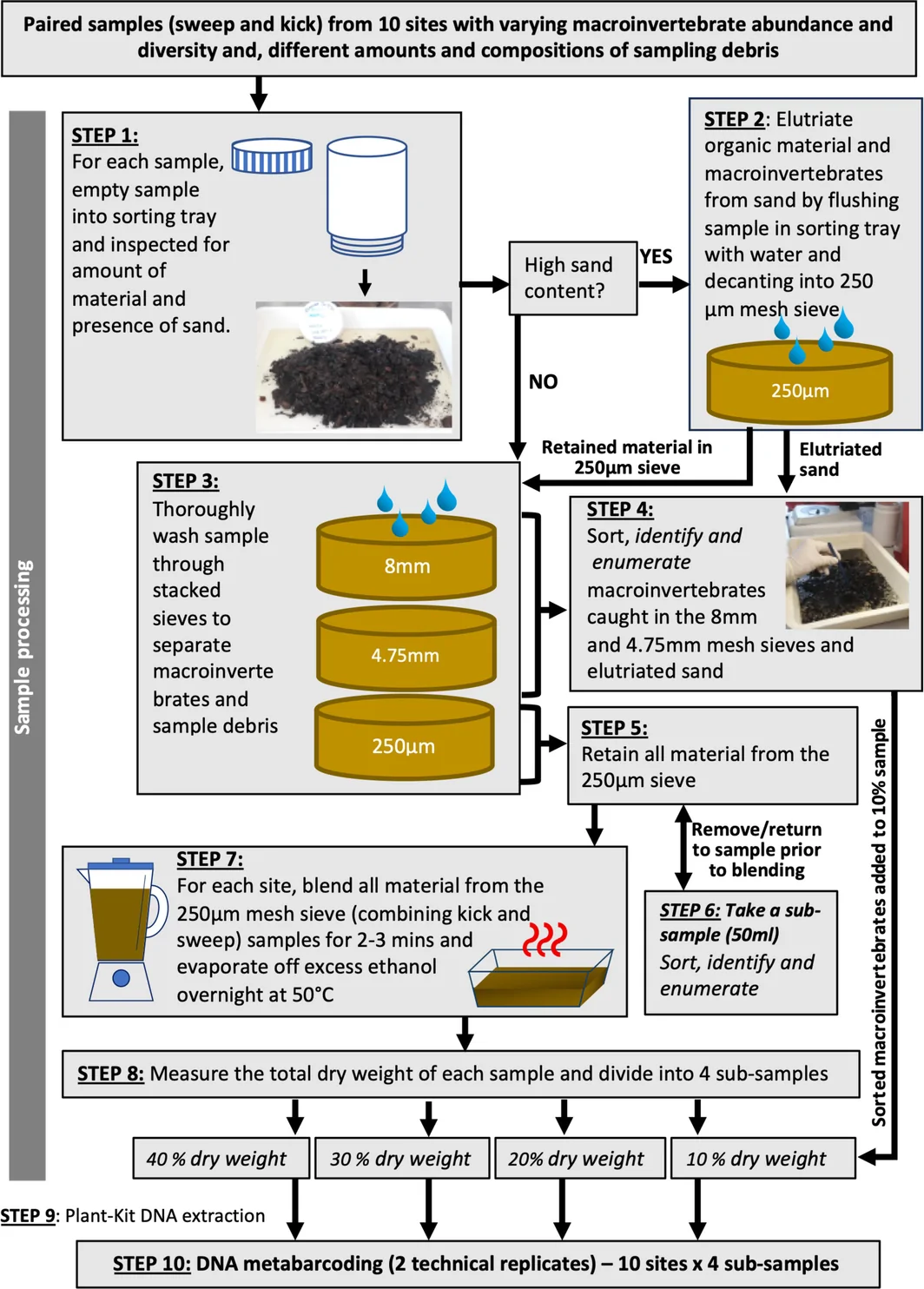
Sample processing workflow used in this study which was designed to reduce the volume of net‐collected material processed for unsorted DNA metabarcoding. The parts of the method presented in italics were used for this study but could be omitted for large scale sample processing.

Elutriated organic material from high sand content samples and all material from other samples were then thoroughly washed with water through stacked 8 mm, 4.75 mm and 250 μm mesh sieves (Figure 1). Care was taken to rub leaves and stones and tease apart algae and plant material to dislodge animals. Sieves were rinsed and inspected for debris and animals, then soaked and washed in hot soapy water and thoroughly rinsed again between samples from different sites. To reduce the volume of material for DNA metabarcoding, material from the 8 mm and 4.75 mm sieves was scanned for any macroinvertebrate taxa which were retained, and the remaining material discarded. All material in the 250 μm mesh sieve was retained, as it was likely to contain the highest abundance of macroinvertebrates. The retained material was stored in absolute ethanol at 4°C until further processing. Sweep and kick samples were stored separately for each site.

Animals sorted from the elutriated sand and the 8 mm and 4.75 mm mesh sieves were identified to bioassessment (mostly family) level and enumerated. In addition, the retained fraction was mixed, and a 50 mL sub‐sample was taken from each sweep and kick sample; all animals were then sorted, identified to bioassessment (mostly family) level, and enumerated for comparison to metabarcoding identifications. Macroinvertebrates and debris from the 50 mL sub‐samples were then returned to the retained material prior to metabarcoding.

Excess ethanol was removed from the retained material and the kick and sweep samples were combined in a glass blender (Laboratory Blender model 8011EG, Waring Commercial, Connecticut, USA) for each site and blended for 2–3 min, ensuring all material was fully homogenised. The blender was rinsed and inspected for debris/animals, then soaked and washed in hot soapy water and thoroughly rinsed again between samples from different sites. Homogenised samples were placed in new 650 mL plastic fast‐food containers (172 mm × 120 mm × 50 mm) and were dried overnight in a convection oven at 50°C until all residual ethanol had evaporated. The total dry weight of each sample was recorded and used to calculate 10%, 20%, 30% and 40% of the total dry weight (Figure 1). These dry weight samples were placed in sterile 50 mL falcon tubes up to the 35 mL mark. If the volume of material exceeded the 35 mL mark, additional tubes were used. Samples were stored in the −80°C freezer until DNA extraction. Animals sorted from the elutriated sand and the 8 mm and 4.75 mm mesh sieve fractions were added into the 10% dry weight tube(s) only to investigate if these taxa appeared in other sub‐samples.

### 2.4 DNA Extraction

Samples were processed using a NucleoSpin Plant II DNA extraction kit (Macherey‐Nagel Inc. Düren, Germany) with a modification of the manufacturer's instructions (Supporting Information 2). For each sample, a buffer containing 2 M NaCl, 20 mM EDTA, 100 mM Tris/HCl, 2% (w/v) CTAB, and 2% (w/v) was added to each 50 mL falcon tube until the dry sample material was fully covered. Three stainless steel beads of 5 mm were added to samples, and samples were then vortexed to thoroughly mix the extraction buffer and dry sample material. Samples were then incubated for 3 h at 65°C, vortexing every hour. If samples had absorbed the buffer, a small additional volume was added to achieve a slurry consistency. After incubation, the 50 mL falcon tubes containing samples were centrifuged for 5 min at 5000*g* and two aliquots of 1 mL of the supernatant were removed and placed in 2 mL tubes. These aliquots were deliberately taken from different parts of the 50 mL tube to ensure that we captured DNA from across the full homogenised volume. For samples requiring multiple 50 mL tubes, we repeated this process for each tube. This approach was chosen to maximise representativeness when working with large, heterogeneous samples. The 2 mL tubes were centrifuged for 5 min at 7000*g* to pellet any remaining sample debris, and 400 μL of clean supernatant was transferred into a 1.5 mL tube for DNA extraction using the NucleoSpin Plant II DNA extraction kit following the manufacturer's instructions. Two DNA extractions were performed from each 50 mL falcon tube, and extractions from the same sample percentage per site were pooled into a single tube prior to PCR.

### 2.5 DNA Metabarcoding

For each site, the four sub‐samples (10%, 20%, 30% and 40%) were treated as a different sample for DNA metabarcoding. We pooled DNA from each site/sub‐sample combination to make a total of 40 metabarcoding samples (10 sites × 4 sub‐samples). These metabarcoding samples were processed with two technical PCR replicates following Elbrecht et al. (2019) using a two‐step PCR process. DNA metabarcoding included negative controls from first round PCR amplifications and a ‘spike control’ of *Scaptodrosophila xanthoreha* (a species found outside the distributional range of our samples used to detect index switching [mistagging]) and a ‘composition control’ (a sample of known composition) to ensure the same species are detected between MiSeq runs, confirming adequate sequencing depth.

The first round PCRs involved amplifying the 3′ end of the mitochondrial cytochrome c oxidase subunit I (COI) DNA barcode region (Hebert et al. 2003) using two overlapping amplicons that were shown to perform well in detecting macroinvertebrate biodiversity from bulk macroinvertebrate samples (Carew et al. 2021). The amplicon used primers BF1/mtCOIintF with BR1/R5 (short amplicon) and BF1/mtCOIintF with dgHCOI/LepR1 (right amplicon) (Table 2). First round PCR primers contained a universal adapter at the 5′ end (Illumina Nextera transposase sequence; Illumina Corporation, San Diego, CA, USA) followed by the target‐specific sequence. First round PCR reactions contained 2 μL of DNA template (1:10 dilution), 8.3 μL molecular biology grade water, 12.5 μL KAPA3G PCR buffer with MgCl_2_ (KAPA Biosystems) with an additional 1 μL MgCl_2_ (25 mM) for a final MgCl_2_ concentration of 2.5 mM, 0.5 μL forward primer (10 μM), 0.5 μL reverse primer (10 μM), and 0.2 μL KAPA3G polymerase (5 U/mL) (KAPA Biosystems) in a total volume of 25 μL, and were amplified using the PCR conditions in Table 2. All PCR amplicons were checked for consistent amplification and size (bp) using gel electrophoresis prior to pooling. The PCR amplicons were pooled based on volume in a ratio 1 (short amplicon):2 (right amplicon) to allow for length‐based biases in amplification in second round PCRs (Table 3). Pooled amplicons were cleaned using ExoSAP‐IT (Applied Biosystems, Waltham, MA, USA) according to the manufacturer's instructions.

**TABLE 2:** Primer pairs, PCR conditions and amplicon sequence lengths (without template primers included) for the primer combinations used in this study.

Amplicon name	Primers		Amplicon length (bps)	PCR conditions	References
	Forward	Reverse			
Right	miCOIintF/BF1	dgHCO2198/LepR1	313	94°C 3 min, followed by 5 cycles of 94°C 30 s, 45°C 40 s, 72°C 60 s followed by 30 cycles of 94°C 30 s, 51°C 40 s, 72°C 60 s, followed by 72°C 5 min	Elbrecht and Leese (2017), Hebert et al. (2004) and Leray et al. (2013)
Short	miCOIintF/BF1	BR1/R5	217	94°C 3 min, followed by 35 cycles of 94°C 40 s, 50°C 30 s, 72°C 60 s, followed by 72°C 5 min	Elbrecht and Leese (2017), Hajibabaei et al. (2012) and Leray et al. (2013)

**TABLE 3:** The dry weight of retained samples used for DNA metabarcoding, the number of 50 mL falcon tubes required for DNA extraction and the dry weight of the unretained material removed by sieving.

Site code	Dry weight of retained sample (g)					No. of 50 mL falcon tubes used				Dry weight of unretained material (g)			
	10%	20%	30%	40%	Total	10%	20%	30%	40%	Organic	Stones	Sand	Total
BNY‐2904‐2	9.3	18.6	27.9	37.2	93.1	1	2	2	3	43.8	597.2	1902	2543
DNG‐31881‐3	1.4	2.7	4.1	5.4	13.5	1	1	1	1	35.7	None	1902	1937.7
DNG‐7957‐4	6.5	13	19.6	26.1	65.2	1	2	2	3	41.2	29.9	194.3	265.4
FEH‐4184‐3	4.4	8.8	13.1	17.5	43.8	1	1	2	2	72.3	203.2	310.3	585.8
FER‐647‐5	3.4	6.8	10.2	13.6	33.9	1	1	1	1	43.4	169	None	212.4
GRD‐7562‐0	1.3	2.6	3.9	5.2	13.1	1	1	1	1	54.6	46	None	100.5
TOO‐4270‐0	7	13.9	20.9	27.8	69.5	1	2	3	3	128.7	None	70.4	199.1
UYT‐199‐3	7.2	14.4	21.6	28.8	72	1	2	3	4	113	348.3	26	487.3
WM1‐232‐0	11.8	23.6	35.4	47.2	118	2	3	4	5	190.4	43	None	233.4
YAR‐3637‐7	8.1	16.2	24.4	32.5	81.2	1	2	3	4	163.6	469	None	632.7

The cleaned pooled amplicons were then used as templates for second round PCRs. Second round primers contained Illumina p5/p7 adapter sequence at the 5′ end, a unique 8 bp index sequence and part of the universal adapter sequence (Illumina Corporation, San Diego, CA, USA) (Supporting Information 3). Primers were synthesised by Macrogen (Seoul, South Korea) and purified using the Macrogen Oligonucleotide Purification Cartridge (MOPC) method, which has similar efficiency to that of HPLC and PAGE purification. Reactions used 3 μL of the pooled first‐round amplicons, 12.5 μL MiFi mix (Bioline, London, England), 2.5 μL forward p5 index primer (10 μM), and 2.5 μL reverse p7 primer (10 μM). PCR conditions were as follows: 94°C for 5 min followed by 12 cycles of 94°C for 30 s, 55°C for 30 s, 72°C for 30 s, then 1 cycle of 72°C for 5 min. Amplicons were pooled in equal amounts, and the library was gel purified using a PureLink Quick Gel Extraction and PCR Purification Combo Kit (Invitrogen, Waltham, MA, USA) and for the final library the concentration was estimated with a Qubit Fluorometer using the dsDNA HS Assay Kit (Life Technologies, Carlsbad, CA, USA). An Illumina MiSeq run using a 600‐cycle flow cell MiSeq sequencing kit V3 (300 bp × 2) (Illumina, San Diego, CA, USA) was performed by the Australian Genome Research Facility Ltd. (AGRF, Melbourne, Australia) to generate sequence data for each sample. Any samples that failed to produce sequence data were re‐run.

### 2.6 Bioinformatics

Bioinformatics processing was performed using QIIME2 (Bolyen et al. 2019) and VSEARCH (Rognes et al. 2016), written using the systemPipeR workflow manager (Backman and Girke 2016) in the R programming language (R Core Team 2025).

A version configured for this experiment is available at https://github.com/mecarew/ten_site_metabarcoding_workflow (Figure S1). Reads were imported into QIIME2 and the demux‐paired function in the Cutadapt plugin (Martin 2011) was used to sort reads into those containing primer sequences. The QIIME2 demux filter function was then used to split reads into amplicon groups (‘short’ and ‘right’) for each site. The trim‐paired function in the Cutadapt plugin was applied to trim primer sequences from reads. The R1 and R2 reads were visualised in quality plots for each amplicon (‘short’ and ‘right’), which were used to determine truncation lengths for DADA2 (Callahan et al. 2016). The DADA2 plugin in QIIME2 was run to perform quality filtering, denoising, merging of paired reads, chimera removal and dereplicating sequences (reads) into amplicon sequence variants (ASVs). As amplicons were shorter than < 320 bp which was shorter than the combined read length (2 × 300 bp), the R1 and R2 reads largely overlapped and were merged to form high‐quality consensus sequences. Error rates were learned using the DADA2 algorithm, and amplicon sequence variants (ASVs) were inferred. Chimeric sequences were identified and removed using the consensus method, resulting in a final table of amplicon sequence variants (ASVs). Low‐abundance and low‐prevalence ASVs were removed in QIIME 2 using abundance–prevalence filtering. ASVs were retained only if they represented 0.0001% and occurred in more than one sample. The LULU algorithm (Frøslev et al. 2017) was used to merge highly similar and co‐occurring ASVs to reduce erroneous diversity due to sequencing artefacts. Alpha rarefaction curves were produced to check that samples contained adequate sequencing depth.

Taxonomic assignment of ASVs was conducted using a two‐step VSEARCH‐based approach. First, VSEARCH was run manually on ASV sequences to determine the percent identity of the top hits for each ASV against a custom amplicon COI reference database (https://github.com/mecarew/DNA_barcode_reference_database_construction) based on invertebrate sequences downloaded from BOLD systems Version 4 and GenBank and also a local reference database. This preliminary step was used to inform the threshold for consensus‐based taxonomic assignment in the subsequent classify‐consensus‐vsearch step, which assigns taxonomy based on the top % identity matches. Hits were retained if they had ≥ 97% sequence similarity and ≥ 80% query coverage, and ASVs with a match < 80% were classified as ‘unassigned’.

As assignments from VSEARCH can contain truncated taxonomic classifications due to taxonomic errors in downloaded sequence data and overlapping interspecific diversity, we constructed a vetted ASV library (https://tools.thewerg.unimelb.edu.au/mwbugs_manual/c6‐tbl_dna.html). An ASV code is consistently assigned to the identical sequence across MiSeq runs, so the same taxonomic classification for that AVS can subsequently be applied whenever that ASV is detected, thereby avoiding the need for repeated reference database searches and correction of classification errors. For this process, we assigned BOLD Barcode Index Numbers (BINs) (e.g., *Orthotrichia* sp. B‐ACN2323) to taxa without species taxonomic identifications, if sequences matched multiple BOLD BINs, we used oldest BIN with the word group (e.g., *Nousia* sp. B‐AAV4108 group), to indicate that an ASV matched multiple BINs and a level of > 97 sequence similarity. For taxa without BIN's we used the GenBank assession codes or BOLD PIDs (e.g., Bdellidae sp. G‐OK042128) or the initials of the person who made the identification (e.g., *Nanocladius* sp. MC‐1). This hierarchical system ensures that all ASVs receive a consistent, traceable label while explicitly flagging taxonomic uncertainty and reducing the risk of over‐splitting or misclassification.

To minimise the impact of potential index‐switching, which can occur more commonly for dominant ASVs, we first filtered ASVs based on their global and per‐sample abundance. Globally rare ASVs (fewer than 5 reads across the dataset) were removed to eliminate remaining singletons and doubletons from our abundance–prevalence filtering. We then applied a scaled proportional filter where each ASV's per‐sample proportion was compared to a threshold calculated based on the ASV's global abundance to favour the removal of index‐switching and not eliminate species detection of rare low read abundance species. ASVs that were high‐frequency globally but present at low proportions in individual samples were removed, as these are likely artefacts arising from index‐switching. The minimum and maximum thresholds for this proportional filter were set to 0 and 0.001, respectively, but can be adjusted depending on evaluation of control samples in different MiSeq runs. We also implemented a species‐based safeguard to account for originating from dominant species not linked to ASV abundance. Species contributing ≥ 5% of the total reads across all samples were considered dominant. Within these dominant species, ASVs with < 11 reads per sample were removed to prevent low‐frequency contaminant ASVs from inflating diversity estimates. The final dataset included ASVs that passed both proportional and dominant‐species filtering, to provide a dataset representing ‘true’ biodiversity while minimising potential sequencing artefacts. Amplicons were kept separate throughout the workflow, with species identifications and read counts combined for diversity analysis. All summaries and visualisations were generated using R (R Core Team 2025).

#### 2.6.1 DNA Metabarcoding Species Detection Across Sub‐Samples

Taxa that were known macroinvertebrates were assigned taxon codes according to Walsh and McCasker (2002). Macroinvertebrate biodiversity in sub‐samples was examined using box and whisker plots and NMDS ordination of community composition (available at https://github.com/mecarew/ten_site_metabarcoding_workflow).

Box and whisker plots were used to visualise the proportion of species richness in each sub‐sample percentage for each site compared to the total species richness for that site; the means, median and interquartile range (IQR) and total range were included in plots. A presence‐absence sub‐sample by species community matrix was created by grouping records by site, sub‐sample and species. An NMDS ordination was then performed using Euclidean distances on the CLR‐transformed matrix, which corresponds to Aitchison distance, as this method is suited to presence–absence data, like that from metabarcoding (Gloor et al. 2017). A two‐dimensional solution was produced using 100 random starts to ensure convergence. Sample scores and metadata were extracted from the final NMDS model and visualised in a scatterplot comparing DNA‐based and morphology‐based community composition across sites.

### 2.7 Comparison of DNA Metabarcoding Identification to Morphological Past Collections

For nine of the ten sites sampled, previous macroinvertebrate samples had been collected and morphologically identified to various taxonomic levels (i.e., species, family, order, class) (Table 1). Due to a high number of morpho‐species which were not linked to interim names from DNA barcoded specimens, we made comparisons at family bioassessment levels with the exceptions of Chironomidae which are identified to subfamily, Acarina to sub order, Oligochaeta and Turbellaria (excluding the Dugesiidae) to class and Nemertea to Phylum. While past sampling included samples as far back as 1993 for some sites, we only included samples taken in the previous 10 years. Presence/absence matrices were constructed separately for the morphological (ten spring samples [as no autumn samples were taken], 2011–2020) and DNA metabarcoding (one autumn sample, 2021) datasets. A site by species community matrix was created by grouping records by site, sample, identification method and species. An NMDS ordination was then performed using Euclidean distances on the CLR‐transformed matrix corresponding to Aitchison distance. A two‐dimensional solution was produced using 100 random starts to ensure convergence. Sample scores and metadata were extracted from the final NMDS model and visualised in a scatterplot comparing DNA‐based and morphology‐based community composition across sites (available at https://github.com/mecarew/ten_site_metabarcoding_workflow).

## 3 Results

### 3.1 DNA Metabarcoding

The final MiSeq library contained 19.2 ng/μL of pooled amplicons and MiSeq sequencing yielded 5,488,171 high‐quality sequence reads with over 76,500 high‐quality sequence reads per sub‐sample for each site (Supporting Information 3). Three sub‐samples in the final dataset were re‐run in subsequent MiSeq runs due to insufficient reads (i.e., 10% FEH‐4184‐3, 30% DNG‐7957‐4 and the 40% BNY‐2904‐2). Raw reads from DNA metabarcoding can be found on the National Centre for Biotechnology's Sequence Read Archive under BioProject PRJNA1369122 (data available at https://www.ncbi.nlm.nih.gov/sra/PRJNA1369122). Accession codes, index combinations, cutadapt, DADA2 and final data summaries can be found in Supporting Information 3. Our spike control was not found in the final data suggested little index switching and our compositional control detected all expected species suggesting sequencing depth was sufficient. A single ASV (131 reads) was detected in one of our negative controls. This ASV was taxonomically assigned to the freshwater golden‐brown flagellate *Poterioochromonas malhamensis*. This taxon did not appear in any samples and is not associated with our study group (macroinvertebrates). We considered it an isolated artefactual detection and not of concern.

Rarefaction analysis showed that sequencing depth was adequate (Figure S2) with the right amplicon requiring a sequencing depth of at least 5000 reads and the short amplicon around 20,000 reads to recover most ASVs in a sample. For each sample we found 71% of ASVs were found in both PCR replicates, 93% if species detections were considered. A total of 3987 (2583 from the short and 1404 from the right) ASVs were found which represented 565 species from 178 families.

Freshwater macroinvertebrates comprised 35% of ASVs and 86% of DNA sequences (reads) returned from DNA metabarcoding, while an additional 16% of ASVs and 5.8% of reads were assigned to other invertebrate groups including terrestrial invertebrates, microinvertebrates and invertebrates whose origin was unknown (Figure 2). We found 11% of ASVs were unassigned (Figure 2A) but these only represented 2.6% of reads (Figure 2B). A similar pattern was observed with other non‐target groups demonstrating that the primers and sieved material were a good source of macroinvertebrate DNA for metabarcoding.

**FIGURE 2:**
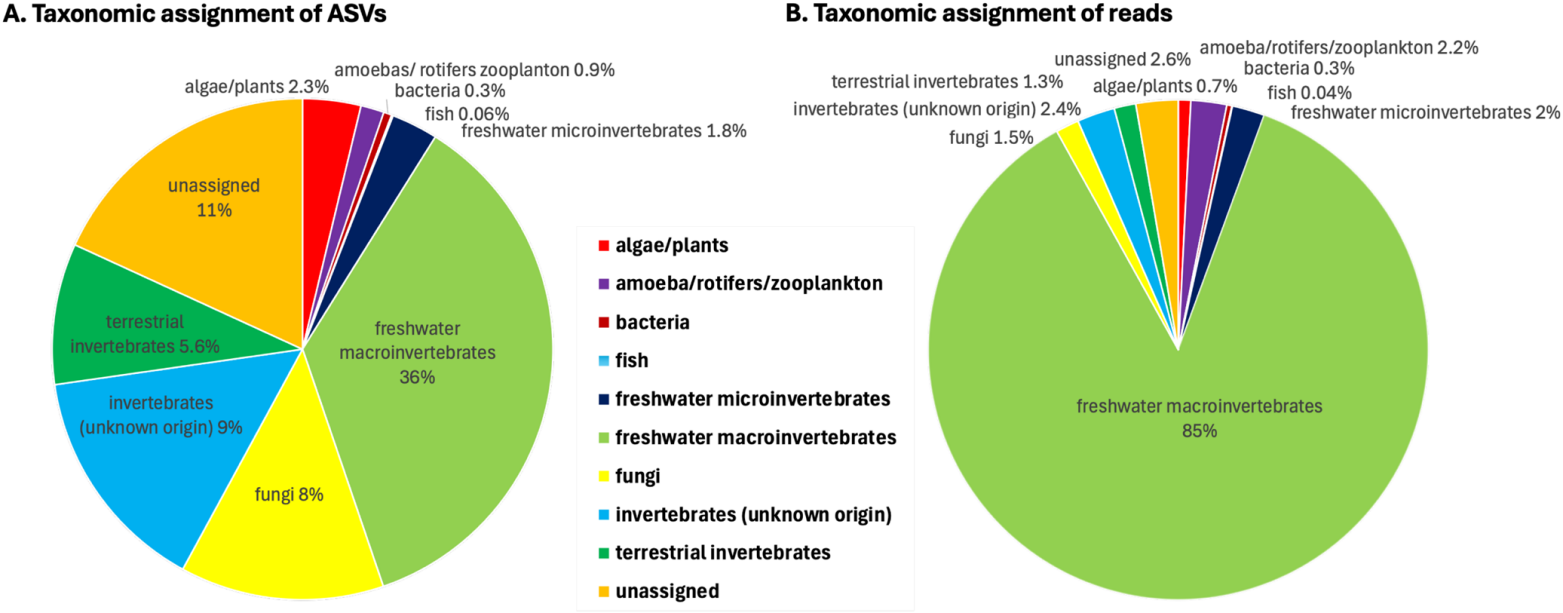
Percentage of amplicon sequence variants (ASVs) (A) and sequence reads (B) returned from DNA metabarcoding for the 10 unsorted samples and their assignment to different taxonomic groups.

Overall, we were able to assign 42% of ASVs and 90% of the reads to species. For freshwater macroinvertebrates, 93% of ASVs representing 99% of sequence reads could be assigned to species. Across the 10 sites, a total of 378 macroinvertebrate species from 85 families were identified.

The sites classified as having ‘high richness’ contained significantly higher numbers of macroinvertebrate species (Welch two‐sample *t*‐test: *t* = 7.55, df = 7.22, *p* < 0.001) and families (Welch two‐sample *t*‐test: *t* = 7.47, df = 7.57, *p* < 0.001) than those classified as ‘low richness’ (Figure 3). No significant difference was observed in species richness between sites with high and low abundance (Welch two‐sample *t*‐test: *t* = −0.17, df = 7.09 *p* = 0.87) or families (Welch two‐sample *t*‐test: *t* = 0.07, df = 7.75, *p* = 0.95) or between abundance class and sample total dry weight (Welch two‐sample *t*‐test: *t* = −1.13, df = 7.19, *p* = 0.29). Overall, more than twice the number of macroinvertebrate species were detected compared to families across most sites (Figure 3).

**FIGURE 3:**
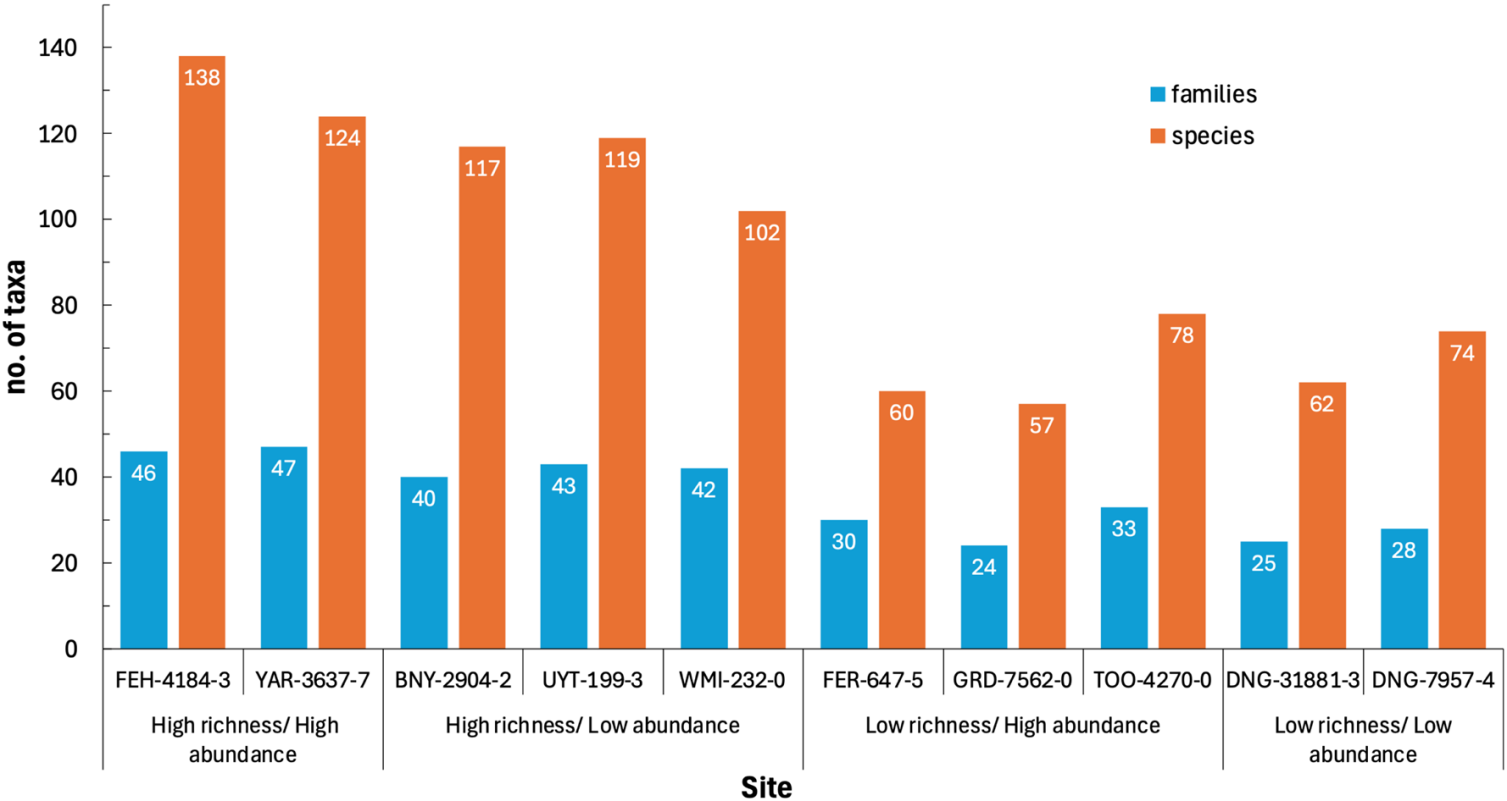
The total number of macroinvertebrate species and families detected from 10 sites across Greater Melbourne using DNA metabarcoding. Sites are classified for abundance and expected richness based on previous surveys (Table 1).

### 3.2 Macroinvertebrate Species Detected With DNA Metabarcoding Across Sub‐Samples

The macroinvertebrate species detected across sub‐samples representing 10%, 20%, 30% and 40% of the dry weight of retained material showed substantial overlap (Figure 4), with no single sub‐sample capturing all species present in the full sample (all sub‐samples combined). Overall, each sub‐sample detected between 67.5% and 96.7% of total species. The greatest variation in species detection occurred in the 10% and 40% sub‐samples, whereas the 20% and 30% sub‐samples were more consistent, detecting 75%–93% of total species. Nevertheless, the proportion of species detected per site did not differ significantly across sub‐sample percentages (one‐way ANOVA: *F*_3_,_36_ = 2.20, *p* = 0.11).

**FIGURE 4:**
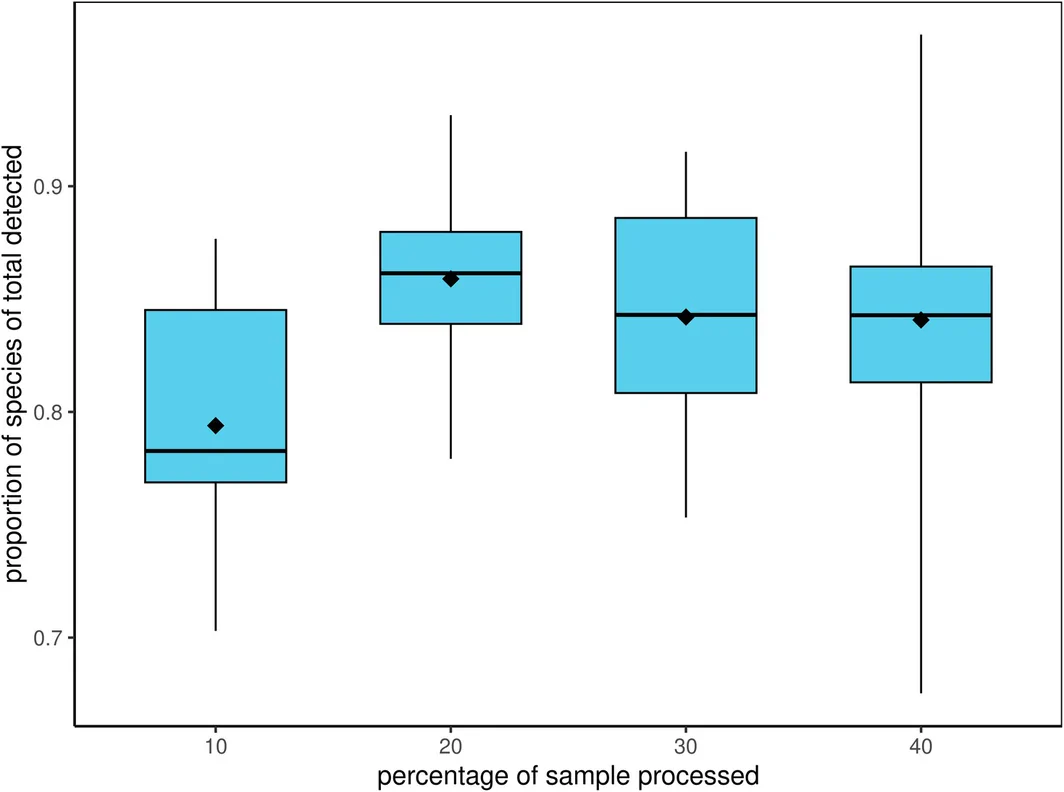
Average proportion of macroinvertebrate species detected across different percentages of dry weight retained (10%, 20%, 30% and 40%) from the 10 sites across Greater Melbourne.

Although variation existed in species detected among sub‐samples, community composition across sites indicated that different sub‐samples from the same site had similar macroinvertebrate communities (Figure 5), suggesting that sub‐sampling did not substantially alter the detected community composition. A low stress value (0.085) indicated that the ordination plot accurately reflected the similarities and differences among sub‐samples.

**FIGURE 5:**
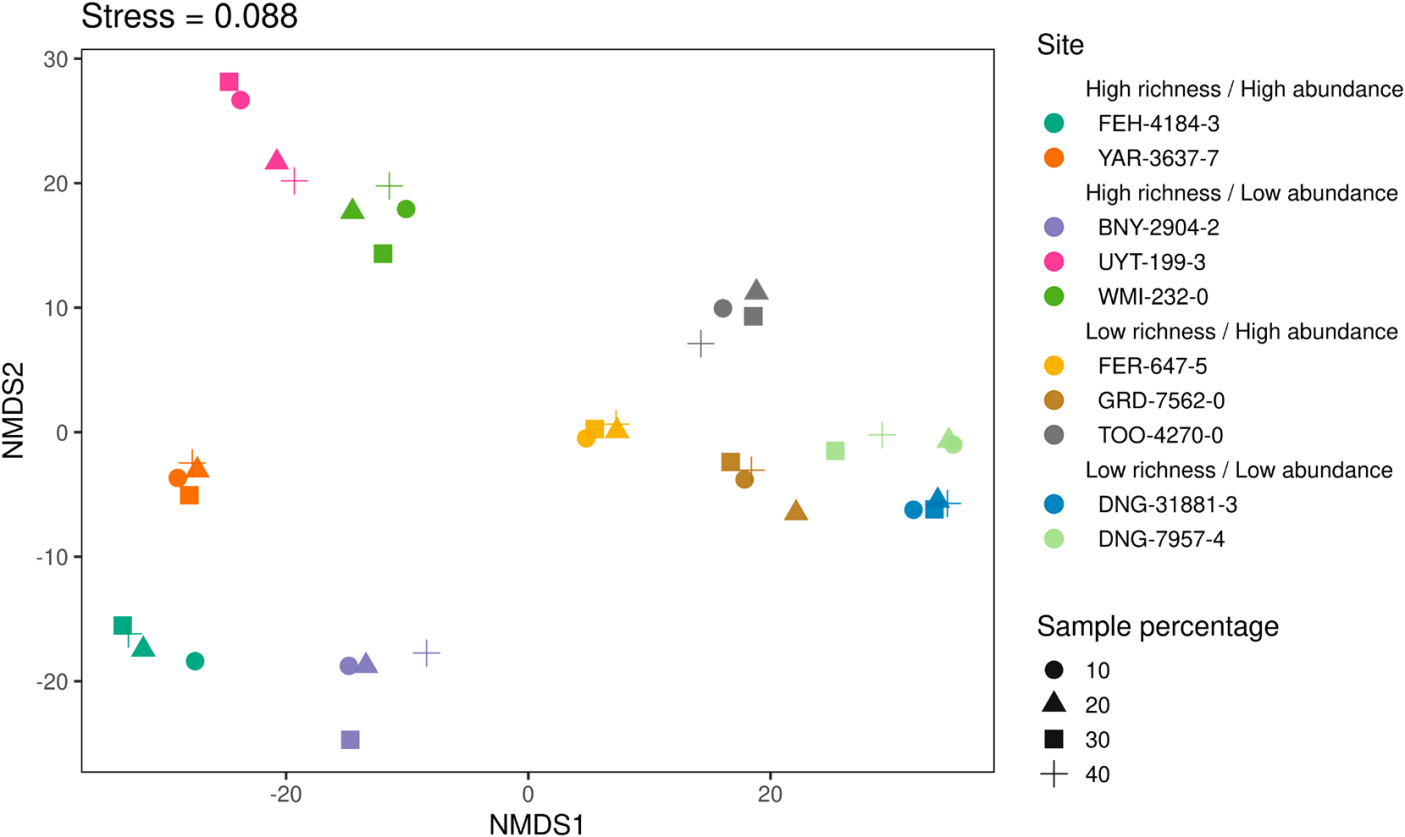
Comparison of community composition when different dry weight percentages of sub‐samples were retained across sites. A presence–absence sub‐sample × species matrix was created by grouping records by site, sub‐sample and species. Non‐metric multidimensional scaling (NMDS) used a centred log‐ratio (CLR)–transformed matrix based on Euclidean distances, equivalent to Aitchison distances. Sites are colour coded, and symbols indicate different sub‐sample percentages.

Macroinvertebrate species richness showed only minimal increases at most sites as more of the sample was processed. However, some samples, such as UTY‐199‐3, BNY‐2904‐2 and TOO‐4270‐0, yielded substantially fewer species in the 40% sub‐sample (Figure 6A). In contrast, low dry weight samples, such as GRD‐7562‐0 and DNG‐31881‐3, exhibited more increases in detected species richness when more than 10% of the sample was processed (Figure 6B).

**FIGURE 6:**
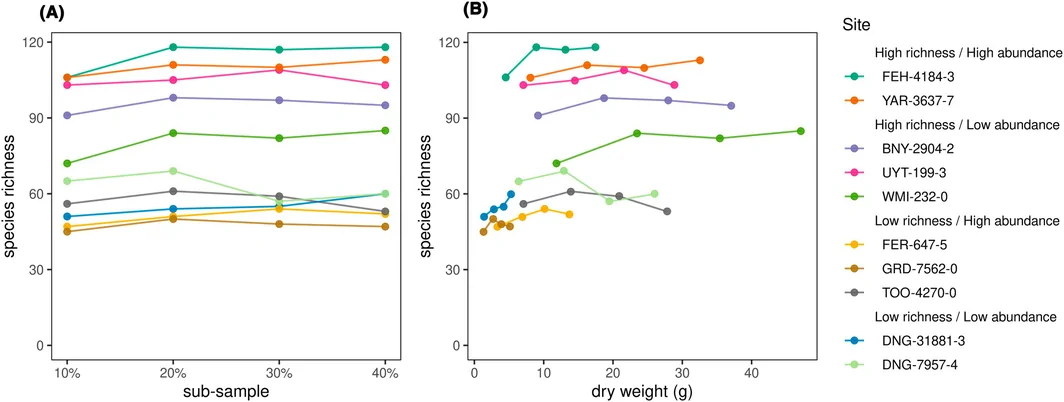
Relationship between species richness and sub‐sampling across sites. (A) Number of species observed in relation to the percentage of dry weights sub‐samples processed (10%, 20%, 30% and 40%) from the total sample. (B) Number of species observed in relation to the corresponding dry weight of the sub‐sample. Points represent observed values and are coloured by site. Lines connect points from the same site.

### 3.3 Impact of Sieving Samples

The number of families and the total abundance of macroinvertebrates found in the unretained material from elutriated sand, the 8 mm and 4.75 mm mesh sieves, and in 50 mL of the retained material from the 250 μm mesh sieve used directly for metabarcoding are shown in Figure 7. The families identified in the 50 mL sub‐samples exhibited a similar pattern of richness estimates (Figure 7) to those obtained from metabarcoding (Figure 3), although fewer families were detected due to the small volume sub‐sampled.

**FIGURE 7:**
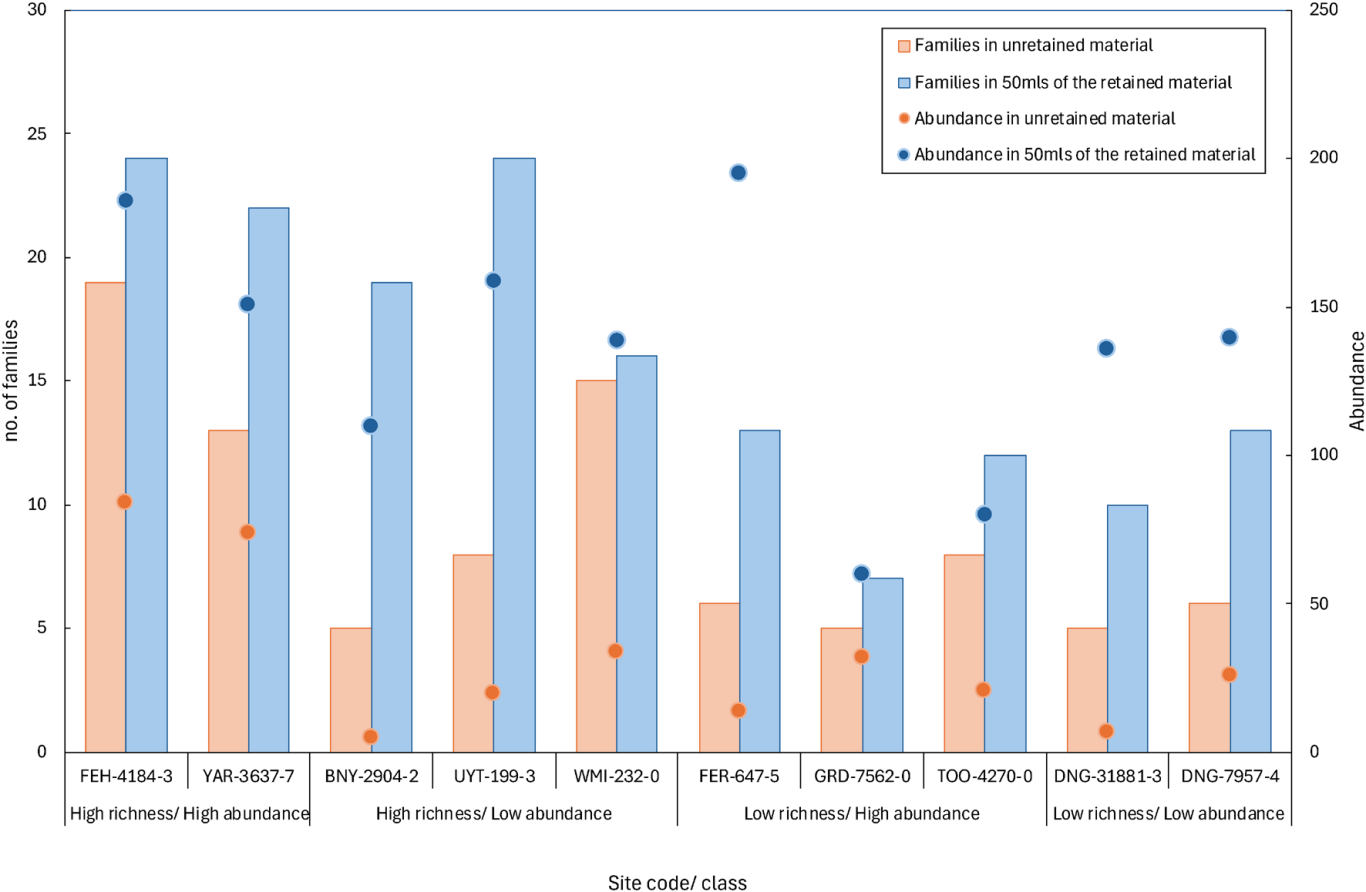
The number of families and the total abundance of macroinvertebrates found in the unretained material (which included elutriated sand and the 8 and 4.75 mm mesh sieves) and in 50 mL of the retained sweep and kick material (from the 250 μm mesh sieve) based on morphological examination.

A small number of taxa (*n* = 3) were present in the morphological samples but not observed in the metabarcoding dataset. These included members of the Dugesiidae (2) and Baetidae (1). It could not be determined whether these taxa were truly absent from the metabarcoding samples or lacked reference DNA barcodes for species‐level assignment.

The unretained material contained animals that could be missed by sieving, as these were trapped in sieves with larger mesh sizes. Our protocol scanned these fractions only for larger taxa, which were then manually added to the retained sample. To test whether these taxa would still be detected if missed during scanning of larger fractions, they were added only to the 10% dry weight sub‐sample from each site. This allowed comparison of taxa found in the unretained material (present only in the 10% sub‐sample) to those detected via metabarcoding in other dry weight sub‐samples. Only three instances occurred in which species present in the larger sieves were not detected in the 20%, 30% or 40% sub‐samples. All three instances were from site FEH‐4184‐3 and included Limoniidae (*Clytocosmus helmsi*), Gripopyteriade (*Leptoperla primitiva*) and Aeshnidae (*Austroaeschna atrata*). All other taxa in larger sieve fractions were detected in the 20%–40% sub‐samples, including very large animals from Gyrinidae, Atyidae and Parastacidae. These taxa were typically detected with high sequence read counts in the 10% sub‐sample and lower counts in the 20%–40% sub‐samples. We also found four instances where a specimen in the large fraction was added to the 10% sub‐samples but was not detected with metabarcoding. These included Hymenosomatidae, Syrphidae and Gordiidae.

The total dry weight of the retained sample ranged from 13.1 to 118 g and samples contained different debris compositions (Table 3, Figure S3). Sieving samples was able to reduce sample dry weights by 69%–99% through removing excess organic material, sand and stones (Table 3). Removal of larger organic material led to a comparative reduction of the dry weight of samples for DNA extraction by 31%–80% per sample.

### 3.4 Comparison of DNA Metabarcoding Identification to Past Collections

We were able to compare family data from past collections for eight of the ten sites which were sampled in the last 10 years (Figure 8). Using an NMDS ordination of community composition, we found that sites characterised through metabarcoding tended to group with sites previously sampled morphologically. This occurred even though those earlier samples were collected in a different season (spring) to the DNA metabarcoding data (autumn). The ordination also revealed a clear separation between sites categorised as high richness (BNY‐2904‐2, FEH‐4184‐3, UYT‐199‐3, WM1‐232‐0, YAR‐3637‐7) to the left side of the plot and those categorised as low richness (DNG‐7957‐4, DNG‐31881‐3, FER‐647‐5, GRD‐7562‐0, TOO‐4270‐0) to the right side of the plot. The low stress value (0.12) of the NMDS plot indicates a good fit of the data and supports the observed site‐based separation, suggesting that metabarcoding recovers similar communities of macroinvertebrates at family level compared to morphologically based identification of samples from previous years, despite samples being collected in different seasons and years.

**FIGURE 8:**
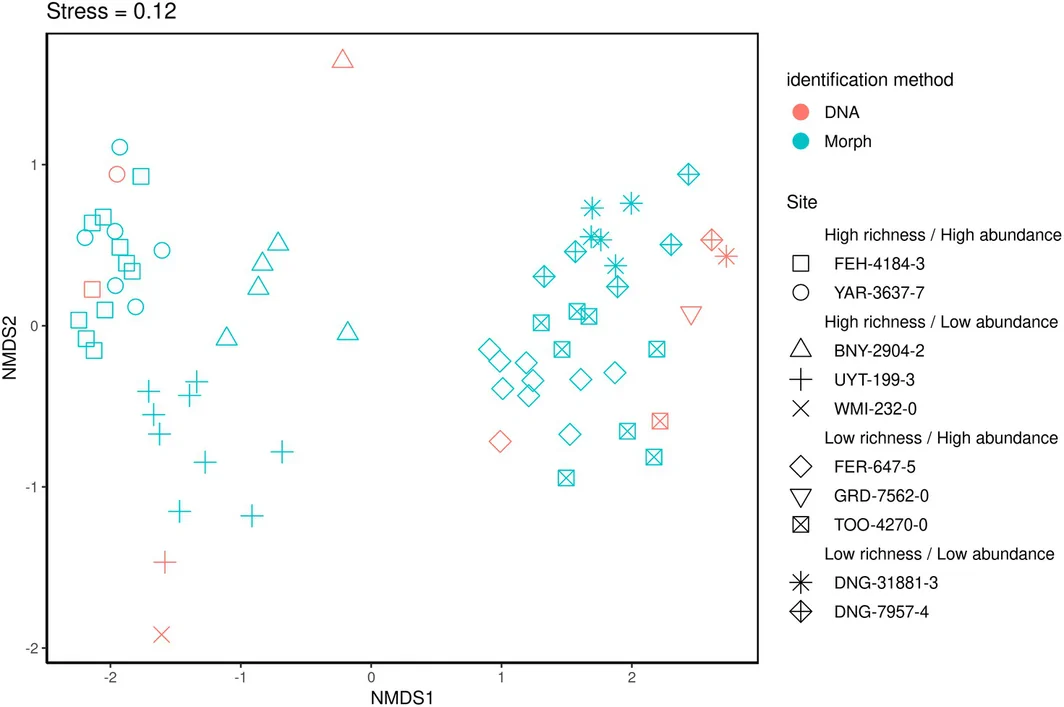
Comparison of macroinvertebrate community composition across sites with historical surveys using morphological (Morph) and DNA metabarcoding (DNA) data based on bioassessment level identifications (mostly family with Chironomidae identified to subfamily, Acarina to suborder, Oligochaeta and Turbellaria (excluding Dugesiidae) to class, and Nemertea to phylum). Non‐metric multidimensional scaling (NMDS) used the centred log‐ratio (CLR)–transformed matrix using Euclidean distances, equivalent to Aitchison distance. Past morphological data was available from eight of the ten spring‐only surveys, from 2010 to 2020 (no data were available for WM1‐232‐0 and GRD‐7562‐0) while the DNA metabarcoding dataset was from one autumn survey (2021).

## 4 Discussion

This study demonstrates a robust, standardised workflow for processing bulk macroinvertebrate samples that contain variable amounts of debris and tissue. By integrating sample processing, DNA extraction, metabarcoding and bioinformatic analysis, our method provides a scalable approach for assessing freshwater biodiversity across sites with differing species abundance, diversity and sample composition.

DNA metabarcoding of unsorted material offers an excellent means to reduce time‐consuming manual sorting from high debris samples typically associated with traditional morphological approaches (Buchner et al. 2021; Macher et al. 2018; Pereira‐da‐Conceicoa et al. 2020). Our approach yielded a high proportion of reads (85%) of macroinvertebrate origin (91% for all invertebrates) using two widely used amplicons that have previously been found to amplify taxonomically diverse invertebrate groups with a low amount of off‐target amplification (< 3% in our study) in unsorted or bulk material (Carew et al. 2021; Elbrecht et al. 2019; Elbrecht and Leese 2017). We found that most species from families that were common and abundant in samples were reliably detected across the different sub‐sample percentages of the dry weight processed, suggesting that sub‐sampling of large volume samples has a modest impact on species detection or the assessment of community structure. This was evident when comparing metabarcoding family identifications to past morphologically identified samples and the similarity of the community structure among sub‐samples from the same site. By evaluating samples with diverse volumes, debris levels, and invertebrate communities, we ensured our approach can consistently detect invertebrates and allow site comparisons across the variety of conditions found in field surveys.

We propose a standardised method for processing macroinvertebrate samples that builds on the rapid bioassessment approach (RBA) where bulk macroinvertebrate samples are collected, combined, then sieved through three entomological sieves with mesh sizes of 8 mm, 4.75 mm and 250 μm. This process reduces the volume of net debris and concentrates macroinvertebrates for DNA metabarcoding. The sieving process removes sand, stones and large wood debris including sticks in net‐collected samples which makes samples suitable for blending. In addition, most current methods evaluated for DNA metabarcoding of unsorted material do not use sieves (i.e., Buchner et al. 2021; Majaneva et al. 2018; Nichols et al. 2019; Pereira‐da‐Conceicoa et al. 2020), but we found that sieving was particularly useful and in turn can reduce costs by reducing the amount of material for DNA extractions. We note that almost all taxa that were in the larger sieves (8 and 4.75 mm) were present in the retained material, with only three instances where a species would have been missed if the unretained material had not been searched. This means that few additional taxa would likely be found by extensively searching unretained material, and that a rapid scan of large conspicuous animals should ensure that few taxa are missed by sieving. Interestingly, large taxa, such as Atyidae and Parastacidae, which were completely removed by sieving were detected with DNA metabarcoding in all sub‐samples, despite only being added to the 10% sub‐sample. This suggests DNA leaching during sample storage (Martins et al. 2019), or else fragments of these large animals that are washed through to the 250 μm sieve.

When sieving material, it is important to ensure most small invertebrates have been washed through to the 250 μm sieve. The first sample we processed (FEH‐4184‐3) had the most macroinvertebrates in the unretained material. After sieving this sample, we increased the time spent sieving, checking that most small taxa had been washed through to the 250 μm sieve. In future use of our method, taxa caught in larger sieves could be directly added to the retained material prior to blending and DNA metabarcoding. However, it would be important to sub‐sample extremely large taxa, such as Parastacidae or Hyriidae, to avoid sequences from these taxa dominating when metabarcoding at the expense of smaller taxa (Elbrecht, Peinert, and Leese 2017).

For our study, we combined kick and sweep samples at the blending stage. However, samples could be kept separate throughout the DNA metabarcoding procedure if information is required on the nature of communities from different habitat types. Alternatively, if this information is unimportant, samples could be combined prior to sieving to save cost and processing time. We found completing sieving steps in batches prior to proceeding to blending was time efficient. One person could potentially sieve 6–10 samples a day, depending on sample volume. We found blending of ethanol‐preserved sample material was highly effective for homogenising them. Buchner et al. (2021) recommended blending unsorted macroinvertebrate kick samples for 3 min, which we also found was suitable, especially for high‐volume samples, producing material composed of a fine powder after blending and drying in the oven. Based on the time taken to blend our samples, which included cleaning the blender between samples, we anticipate that one person could blend at least 10–15 samples per day.

Combining kick and sweep samples, even after sieving, resulted in a large amount of material (up to 118 g) for DNA extraction. While DNA extraction methods for large volumes of unsorted material have been explored (e.g., Buchner et al. 2021; Carew et al. 2021; Macher et al. 2018; Pereira‐da‐Conceicoa et al. 2020; van der Lee et al. 2025), many use soil‐based kits. In contrast, plant DNA extraction kits typically have fewer steps and are more cost effective than those for soil and are suitable for unsorted samples (Majaneva et al. 2018). Therefore, we developed a plant extraction method to deal with large volumes and combined this with the KAPA3G Plant PCR Kits (KAPA Biosystems) for first round PCR. The KAPA3G polymerase is tolerant to plant‐derived PCR inhibitors and can amplify from crude extraction and plant material (Roche 2020), so is well suited to unsorted samples.

Understanding the amount of material to process from large volume samples to assess biodiversity is important. If too little is processed, biodiversity may not be adequately measured. If too much is processed, the method can become unfeasible and costly and not substantially improve biodiversity estimates. Our study showed similar community structure and no significant difference between 10%, 20%, 30% and 40% sub‐samples. Between 75% and 100% of species in the entire sample were detected in sub‐samples. However, we did notice differences for some sites. For example, 10% of dry weight in low volume samples tended to detect fewer taxa compared to high percentage sub‐samples. This suggests that a minimum weight may be required for processing combined kick‐sweep samples which are commonly characterised by high and varying volumes of debris and low ratios of animals to debris compared to kick net samples. While Buchner et al. (2021) showed that as little as 1% of kick samples could detect the majority of biodiversity, further testing of low volume kick‐sweep samples should be completed to determine if a minimum sample dry weight should be applied. The 20% sub‐samples, and to a lesser extent the 30% sub‐samples, tended to yield the higher average biodiversity with less variability and should be further explored as a routine sub‐sample size for high volume samples. Interestingly, at multiple sites, 40% sub‐samples showed higher variation and detected fewer species on average than 20%–30% sub‐samples, suggesting processing large sub‐samples may not be optimal. However, with only ten samples, it is difficult to determine whether this pattern reflects true extraction differences or variation in extractions. To further improve our approach, the inclusion of blank controls at the blending and DNA extraction stages could be added to evaluate potential cross‐contamination between samples. In addition, using dual indexing could reduce potential index‐switching. Our study has also shown that it is important to evaluate samples from sites with differing richness, abundance and amount and type of samples debris to calibrate the optimal amount of sample to process.

### 4.1 Concluding Remarks

DNA metabarcoding can reduce aquatic macroinvertebrate sample processing times and costs, as well as provide fine resolution taxonomic data for broad‐scale biomonitoring programmes to support the prioritisation of waterway health management and conservation. We developed a workflow to handle complex samples and implemented a bioinformatic pipeline to ensure reproducible analyses across multiple users. Our approach provides high confidence in biodiversity assessments of macroinvertebrate communities derived from complex samples and is paired with a sample processing and metabarcoding protocol that can be readily adapted to a wide range of invertebrate samples where organisms are mixed with sampling debris. Although our study focused on aquatic macroinvertebrates, the methods we developed, including the sample processing workflow and reproducible bioinformatic pipeline, are directly adaptable to other invertebrate samples containing mixed debris, making the approach highly adaptable for biodiversity monitoring across a range of taxa and habitats.

## Author Contributions

M.C. wrote the manuscript and designed the experiment, completed the laboratory work and data analysis. J.C. wrote the bioinformatic workflow for the metabarcoding data. G.H. collected the samples in the field and worked with M.C. to design the sieving protocol. A.H. and R.C. assisted in the experimental design, data analysis and writing the manuscript.

## Funding

This work was supported by the Australian Research Council (LP200100381) with additional support from Melbourne Water.

## Ethics Statement

All sampling was conducted in accordance with relevant Australian legislation and regulations governing the collection of freshwater macroinvertebrates. Samples were collected under the relevant permits issued by Parks Victoria, AA‐0241308. Sampling procedures were designed to minimise impacts on organisms and their habitats. No animals were experimentally handled or subjected to procedures requiring animal ethics approval.

## Conflicts of Interest

The authors declare no conflicts of interest.

## Supporting information


**Figure S1:** An overview of the GitHub metabarcoding workflow set out as a series of linear notebooks. The workflow integrates both *R* + QIIME2 to process raw MiSeq reads including adaptor and primer trimming, DADA2 denoising, LULU curation, and VSEARCH‐based taxonomic assignment using custom reference libraries. Final scripts merge amplicon sequence variants (ASV) tables, generate rarefaction curves, and apply PCR‐replicate and contamination filtering to produce a vetted dataset for downstream analyses.

**Figure S2:** Alpha rarefaction curves were generated to assess the sequencing depth and species richness across samples.

**Figure S3:** Examples of the different compositions of unprocessed samples prior to sieving from four sites: (A) GRD‐7562‐0 (a low volume sweep), (B) WM1‐232‐0 (a high‐volume sweep), (C) YAR‐3637‐7 (a high sand kick) and (D) FER‐647‐5 (a high algae/aquatic plant kick). The GRD‐7562‐0 sample (A) with the lowest dry weight consisted of very little organic matter mostly from introduced trees, while the WM1‐232‐0 with the highest dry weight contained high amounts of organic matter from the surrounding native trees and shrubs (B). Sites such as YAR‐3637‐7 (C) contained high amounts of sand in addition to organic matter and required elutriation, while sites such as FER‐647‐5 (D) contained large amounts of algae and aquatic plants.

**Supporting Information: 1.** Bulk net‐contents sample processing manual.

**Supporting Information: 2.** DNA extrac+on from unsorted invertebrate samples manual.

**Supporting Information: 3.** Miseq information.

## Data Availability

Raw reads from DNA metabarcoding can be found on the National Centre for Biotechnology's Sequence Read Archive under BioProject PRJNA1369122 (data available at https://www.ncbi.nlm.nih.gov/sra/PRJNA1369122). All scripts used to process data, including bioinformatic and statistical analysis for this study, can be found at https://github.com/mecarew/ten_site_metabarcoding_workflow. The original script adapted for this manuscript can be found at https://github.com/pearg/metabarcoding_workflow. Copies of sample details, outputs and the final dataset can be found in Supporting Information 3. We have also included manuals in Supporting Information 1 and 2 that outline the sample processing and DNA extraction in detail.
