# Supplementary material for: Simplifying Macroinvertebrate Biodiversity Assessment: A Standardised Metabarcoding Method for Invertebrate Samples With High Volumes of Debris
Source: Mol Ecol Resour. 2026 Oct 4;26(7):e70213. doi: 10.1111/1755-0998.70213 (PMC13635761; doi:10.1111/1755-0998.70213)
Supplement: Supplementary file 1 [file MEN-26-e70213-s001.pdf]

A

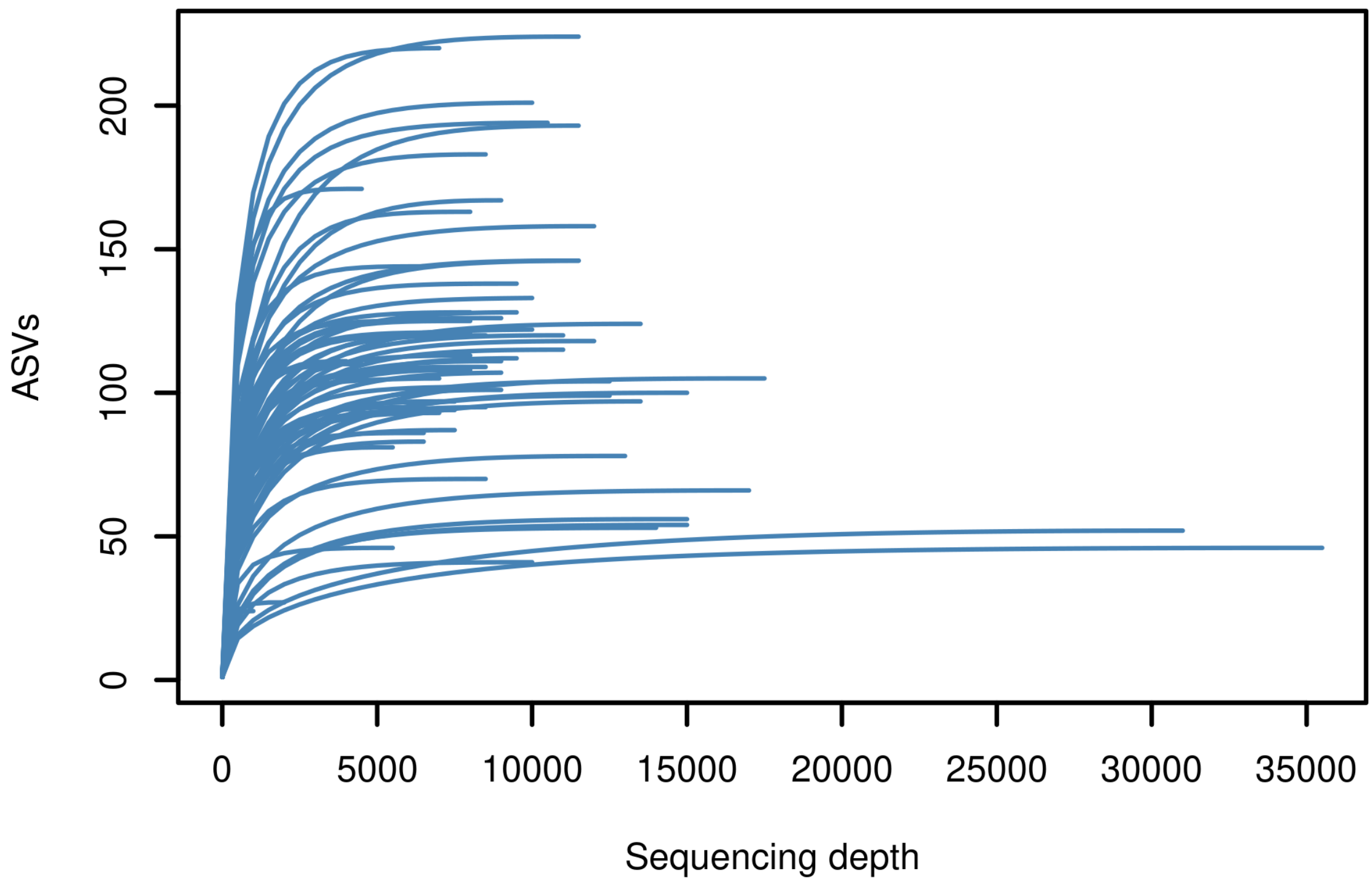
ASVs
0
50
100
150
200
0
5000
10000
15000
20000
25000
30000
35000
Sequencing depth

B

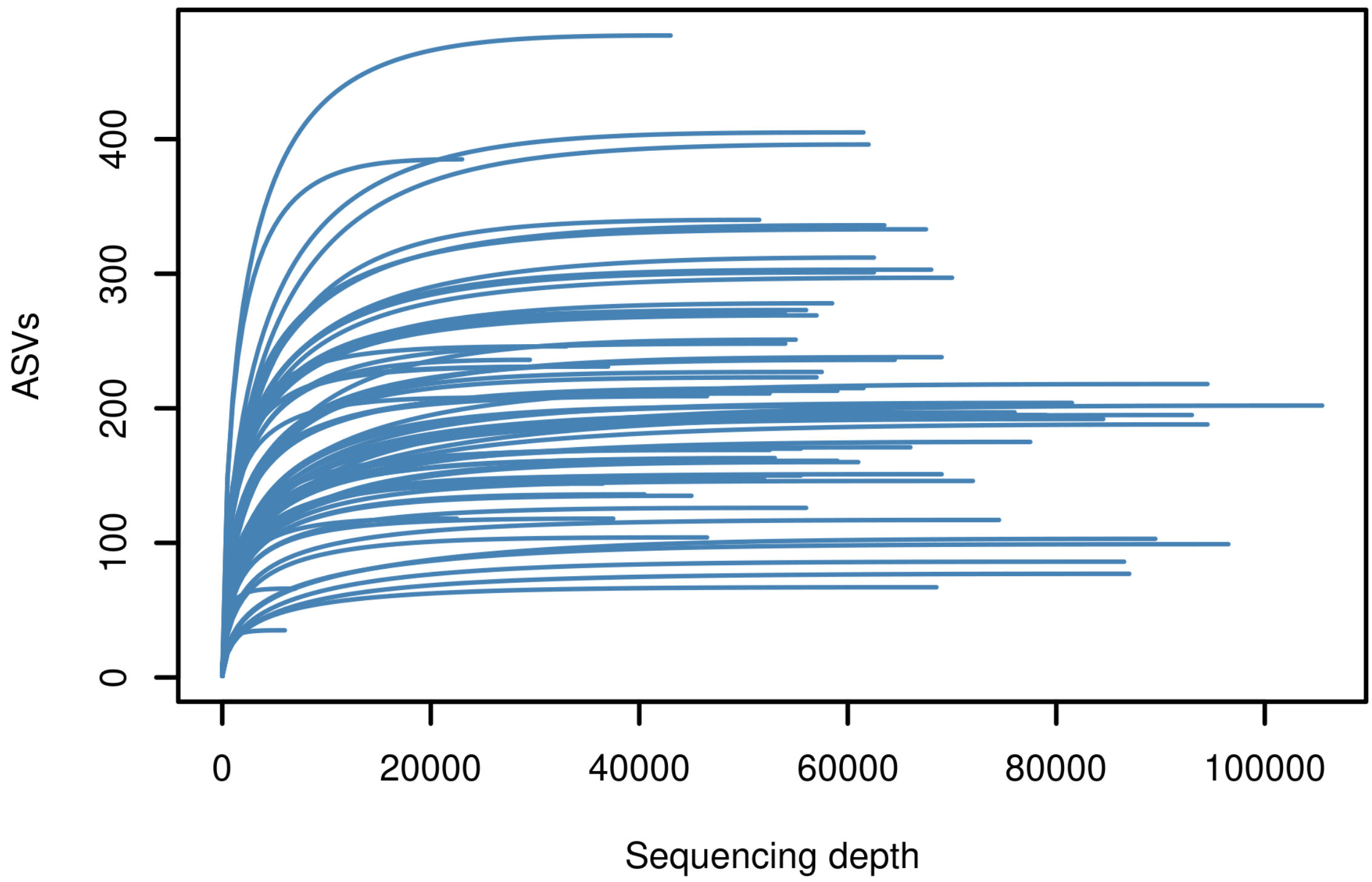
ASVs
0
100
200
300
400
0
20000
40000
60000
80000
100000
Sequencing depth
