# Supplementary material for: Simplifying Macroinvertebrate Biodiversity Assessment: A Standardised Metabarcoding Method for Invertebrate Samples With High Volumes of Debris
Source: Mol Ecol Resour. 2026 Oct 4;26(7):e70213. doi: 10.1111/1755-0998.70213 (PMC13635761; doi:10.1111/1755-0998.70213)
Supplement: Supplementary file 2 [file MEN-26-e70213-s002.pdf]

DNA extraction from unsorted invertebrate samples manual

Overview

This document is a detailed overview of the DNA extraction method used for processing unsorted freshwater invertebrate samples from the 'Bulk net-contents sample processing manual' for DNA metabarcoding. It can be adapted for use in for any large volume samples.

Materials/Equipment:

- Macherey-Nagel plant II DNA extraction kit
- Sieved, blended and dried invertebrate samples in 50ml tubes (stored in -80°C freezer)
- 1.5 mL microcentrifuge tubes
- 2 mL microcentrifuge tubes
- Disposable gloves
- Centrifuge
- Vortex mixer
- Ethanol (100%)
- 8mm stainless steel balls

SOP training required:

- Centrifuge training
- Ethanol training
- Autoclave training
- Weighing out CTAB (Appendix 1)

Before beginning:

- Check the number of extractions left in the kit.
- If starting a new kit, you'll need to add ethanol to PW1
- Buffer will need to be autoclaved
- YOU WILL NEED FULL PPE (including a
- Make up extraction buffer:

Extraction buffer	Amount to add	
2M NaCl	116.8g	Weigh solid
20mM EDTA	40mL	0.5M stock (solution)
100mM Tris/HCl pH8	100mL	1M stock(solution)
**2% (w/v) CTAB[1]**	20g	Weigh solid
2% (w/v) Polyvinylpyrrolidone (MW 40,000)	20g	Weigh solid
	make up 1L with ddH_20^*	

[1]Please follow instruction in Appendix 1 for weighing CTAB

[2] It is best to add less ddH_20 before autoclaving then top up after to 1L (Ensure it is in a solid tray in the autoclave)

DNA extraction protocol overview

1	**Defrost samples**	Retrieve samples from -80°C		
2	**Cell lysis**	Add extraction buffer and a steel ball, vortex.<br>Incubate at 65 °C 3hr (vortexing frequently)<br>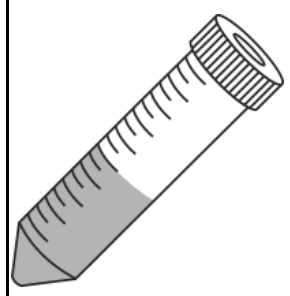		
3	**1st separation of supernatant**	Centrifuge at 5000 rpm for 5 mins<br>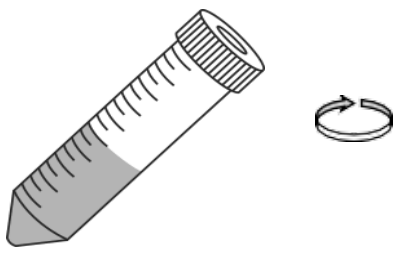		
4	**2nd Separation of supernatant**	Transfer 1.5 ml of supernatant to a 1.5 ml microcentrifuge tube<br><br>spin 11,000 x *g,* 3 mins<br>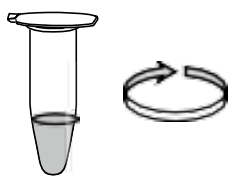		
5	**Filtration / Clarification of lysate (purple column)**	11,000 x *g*, 2 min *(x 2 1.5ml tubes per 50ml tube* ***transfer 410 µL****)*<br>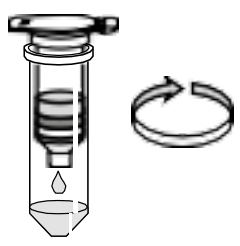		
6	**Adjust DNA binding conditions**	450 µL PC (mix by pipetting gently up and down)		
7	**Bind DNA (green column)**	11,000 x *g*, 1 min<br>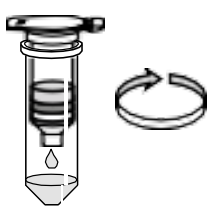		
8	**Wash and dry silica membrane**	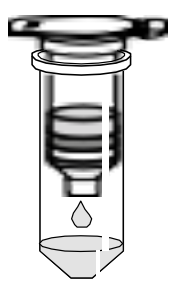	1st 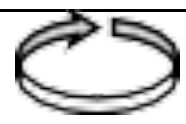	400 µL PW1<br>≥ 11,000 x *g*, 1 min
			2nd 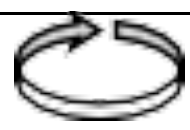	700 µL PW2<br>≥ 11,000 x *g*, 1 min
			3rd 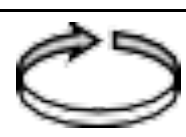	200 µL PW2<br>≥ 11,000 x *g*, 2 min
9	**Elute DNA**	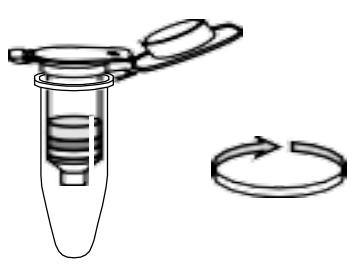	50 µL PE<br>65 °C, 5 min<br>≥ 11,000 x *g*, 1 min<br>------------------------------<br>Repeat elution step	

Extended protocol:

1. **Defrost samples**
   - Turn water bath on to 65°C and (place PE buffer in water bath to heat to 65°C).
   - Do extractions in batches of 12 x 50ml tubes at a time ensuring that all tubes from a site are in a batch. I tend not to do samples with similar codes in the same batch minimise possible mix-ups.

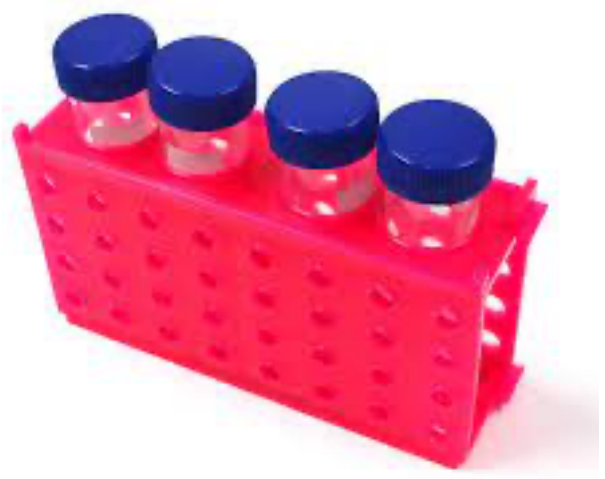

2. **Cell lysis**
   - Add extraction buffer (enough to cover material) and a steel ball each 50mL tube and vortex thoroughly to ensure buffer and sample are well mixed. Make sure lids are on securely. Incubate at 65°C for 3 hrs, vortexing every 30mins. Also, check if buffer needs topping up and add where required.

3. **1st separation of supernatant**
   - After incubation, place 50ml tubes in the large centrifuge with the 50mL rotor and spin 5000 rpm for 5 mins. You'll need to balance the rotor by placing tubes of similar weights opposite each other (or use balances). The centrifuge will not run if not properly balanced.
   -

4. **2nd separation of supernatant**
   - Separate out 1.5 mL of supernatant from each 50 mL tube and place in a 1.5 mL microcentrifuge tube. Label tubes with an abbreviated site code (e.g., TAR or WER etc)
   - Spin 11,000 x *g,* 3 mins in the microcentrifuge

5. **Filtration / Clarification of lysate (purple column)**
   - For each 50ml tube, do **two** DNA extractions.
   - Transfer 410 µL to each into the purple column held in 2 mL microcentrifuge tube (x2).

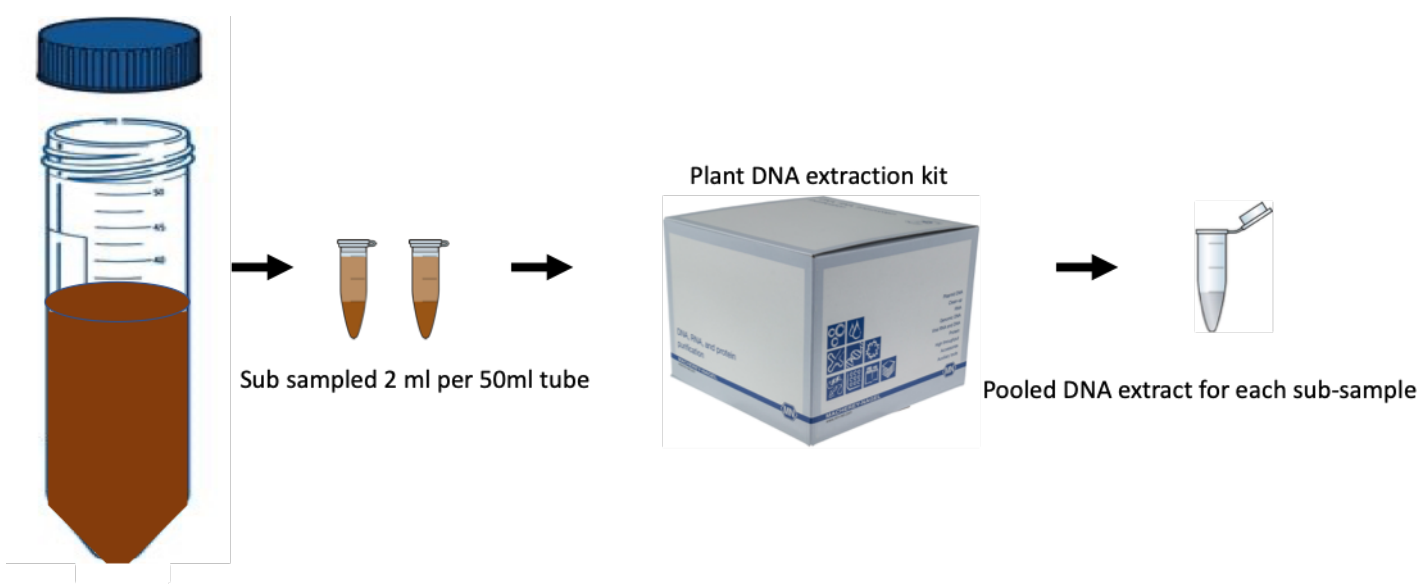

   - Spin at 11,000 x *g,* 2 mins in the microcentrifuge

- You do not need to label the column but label the lid of the 2 mL tube, this is where the DNA will be. The purple column is discarded after the spin.

6. **Adjust DNA binding conditions.**
   - Add 450 µL of the PE binding buffer (from the kit) to the supernatant and mix well by pipetting up and down several times.

7. **Bind DNA (green column)**
   - Place a green DNA binding column (provided in the kit) into a new collection tube.
   - Label lid of green DNA binding column.
   - Add the entire sample volume (approximately 700 µL) to the column.
   - Spin at 11,000 x *g,* 1 min in the microcentrifuge.
   - Discard the flow-through collection tube into DNA extraction waste.
   - place the column back into a new collection tube.

8. **Wash and dry silica membrane**
   - Add 400 µL of the provided PW1 washing buffer I to the column.
   - Spin at 11,000 x *g,* 1 min in the microcentrifuge.
   - Keep the column but discard the flow-through into a Schott bottle for discard.
   - Repeat the washing step with 700 µL of PW2 washing buffer II*.
   - Spin at 11,000 x *g,* 1 min in the microcentrifuge.
   - Keep the column but discard the flow-through into a Schott bottle for discard.
   - Repeat the washing step with 200 µL of PW2 washing buffer II.
   - Spin at 11,000 x *g,* 2 mins in the microcentrifuge.

*Double check required ethanol has been added

9. **Elute DNA**
   - Transfer green DNA binding column to a clean labelled (with the full site/sample information microcentrifuge tube.
   - Add 50-100 µL of elution buffer (provided in the kit) warmed to 65°C directly onto the column membrane.
   - Incubate at room temperature for 5 minutes.
   - Spin at 11,000 x *g,* 1 min in the microcentrifuge
   - Pool DNA replicates from the same site/sample into a fully labelled single tube.
   - Store the extracted DNA at -20°C or proceed with downstream applications.

Appendix 1. Standard Operating Procedure (SOP): Weighing Cetyltrimethylammonium Bromide (CTAB)

1. Purpose

To outline the safe and accurate method for weighing Cetyltrimethylammonium Bromide (CTAB) for preparation of molecular biology reagents and DNA extraction buffers.

2. Scope

This SOP applies to all laboratory personnel weighing CTAB.

3. Safety and PPE

CTAB is harmful if inhaled, swallowed, or in contact with skin and eyes.

Required PPE

- Laboratory coat
- Nitrile gloves
- Safety glasses
- **Disposable particulate mask (P2/N95) or equivalent to prevent inhalation of CTAB dust**

Engineering Controls

- Weigh CTAB in a well-ventilated area.
- Avoid creating dust during handling.

4. Materials and Equipment

- Cetyltrimethylammonium Bromide (CTAB)
- Analytical balance (±0.001 g or better)
- Weigh boat or weighing paper
- Spatula
- Labelled container or buffer vessel

5. Procedure

1. Put on all required PPE, including mask.
2. Ensure the analytical balance is clean, level, and calibrated.
3. Place a clean weigh boat on the balance and tare to zero.
4. Using a clean spatula, slowly add CTAB to the weigh boat.
5. Add material carefully to avoid dust generation.
6. Adjust quantity until the required mass is reached.
7. Record the mass in the laboratory record.
8. Transfer CTAB carefully to the designated container or solution.

6. Clean-up and Waste Disposal

1. Return unused CTAB to its original container and seal tightly.
2. Dispose of contaminated weighing materials according to chemical waste procedures.
3. Clean balance and surrounding surfaces.
4. Remove PPE and wash hands thoroughly.

7. Documentation

- Record date, mass weighed, purpose, and operator initials.
- Record CTAB lot number if required.

8. References

- CTAB Safety Data Sheet (SDS)
- Institutional laboratory safety guidelines
