# Supplementary material for: Simplifying Macroinvertebrate Biodiversity Assessment: A Standardised Metabarcoding Method for Invertebrate Samples With High Volumes of Debris
Source: Mol Ecol Resour. 2026 Oct 4;26(7):e70213. doi: 10.1111/1755-0998.70213 (PMC13635761; doi:10.1111/1755-0998.70213)
Supplement: Supplementary file 3 [file MEN-26-e70213-s003.pdf]

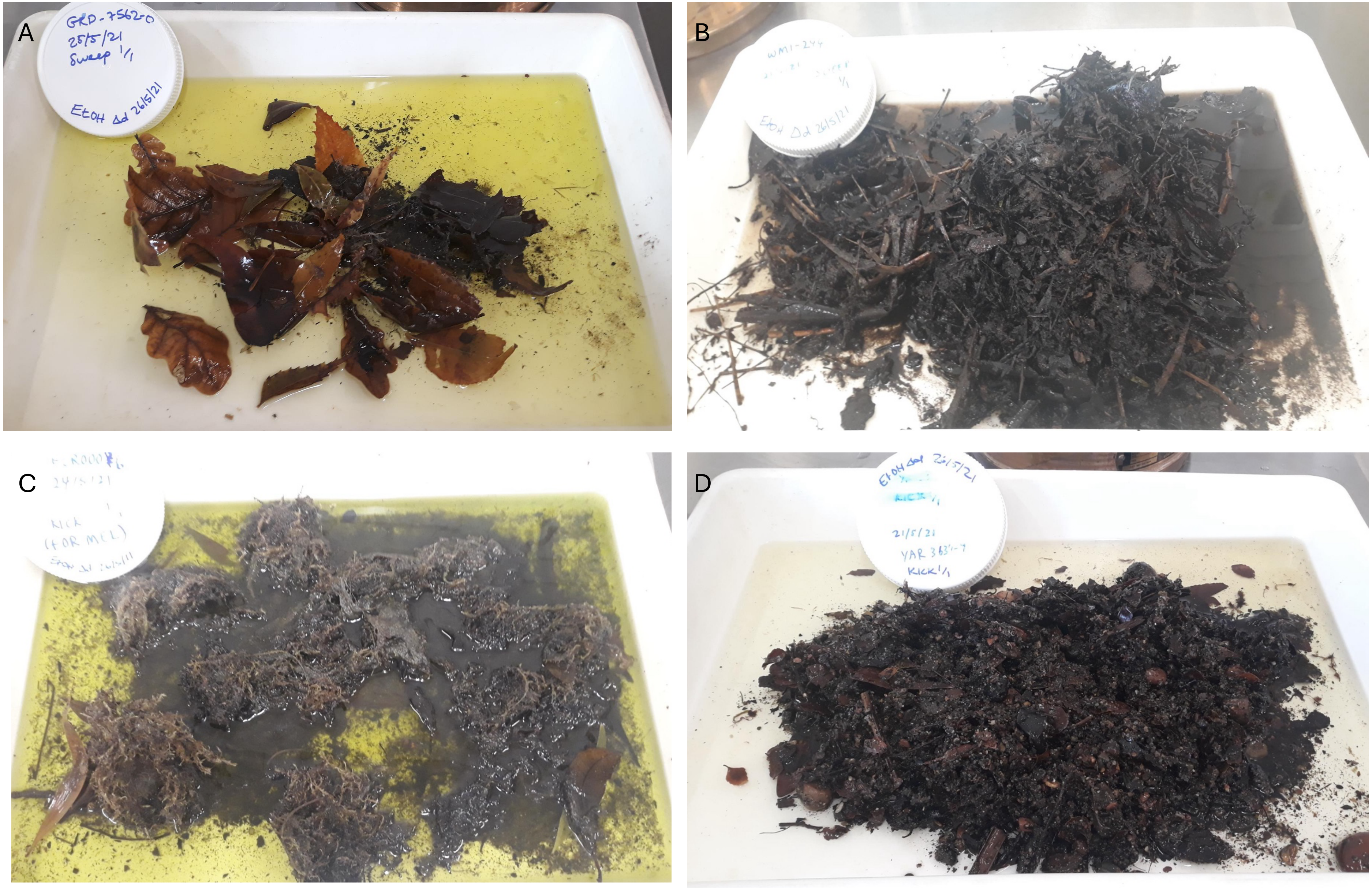
A
GRD-75620
25/5/21
Sweep 1/1
EtOH Ad 26/5/21
B
EtOH Ad 26/5/21
C
KICK
D
21/5/21
KICK 1/1
