# Supplementary material for: Simplifying Macroinvertebrate Biodiversity Assessment: A Standardised Metabarcoding Method for Invertebrate Samples With High Volumes of Debris
Source: Mol Ecol Resour. 2026 Oct 4;26(7):e70213. doi: 10.1111/1755-0998.70213 (PMC13635761; doi:10.1111/1755-0998.70213)
Supplement: Supplementary file 4 [file MEN-26-e70213-s005.pdf]

# Bulk net-contents sample processing manual

***Developed by Melissa Carew and Gen Hehir***

## Overview

This document provides a detailed overview of the laboratory processing of freshwater invertebrate samples for DNA metabarcoding. Two samples are collected using rapid bioassessment protocols (kick-and-sweep or sweep-and-sweep) and processed together. It describes the procedures undertaken after sample collection, including sieving, drying, blending, and weighing, which have been optimised for DNA-metabarcoding workflows. Please refer to the *DNA Extraction Manual for Bulk Material* for the subsequent steps involved in processing these samples for DNA metabarcoding.

## Equipment

- 3-4 white sorting trays
- 250µm sieve (250mm diameter)
- 4.75mm sieve (250mm diameter)
- 8mm sieve (250mm diameter)
- An extra sieve of the same diameter (large mesh size to ensure bottom sieve is not on bottom of sink)
- Tweezers/ plastic pipettes
- 5 mL vials (for taxa in non-retained fraction)
- Large bucket
- Lab coat and safety glasses

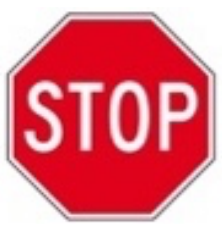


Ensure you have completed the appropriate training for working with ethanol and are aware of hazardous steps performing this protocol (this includes wearing an apron/lab coat and safety glasses/ear protection

## Method overview:

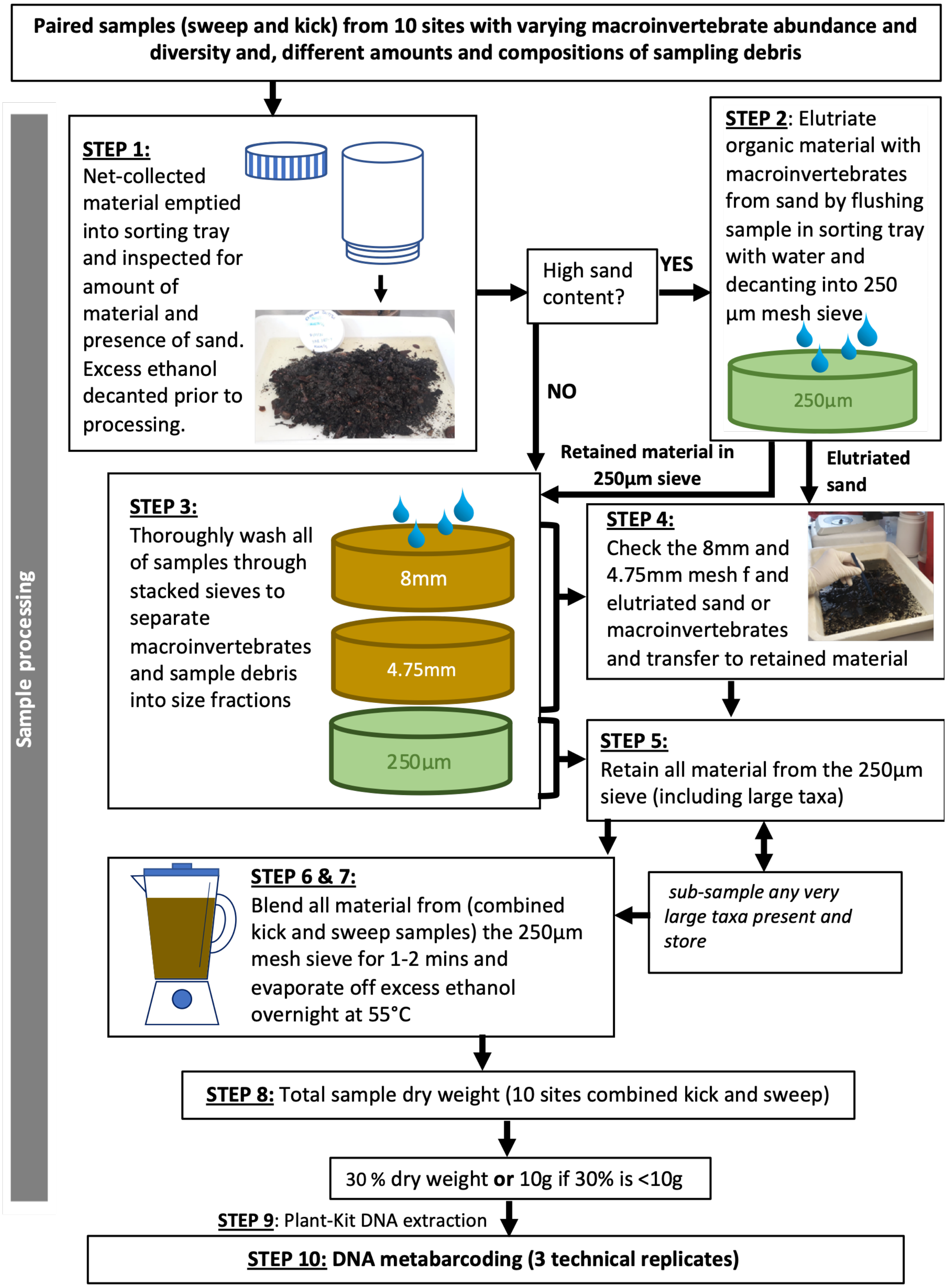

## STEP 1: Inspected sample

- Empty sample from white container into sorting tray.
    - Does the sample contain a lot of sand? - see Step 1a: Note: In some instances, samples will be elutriated in the field too. This will be indicated on the white sample container. The elutriated sand should be checked for animals and does not need to be sieved.
- Take a photograph of sample for future reference

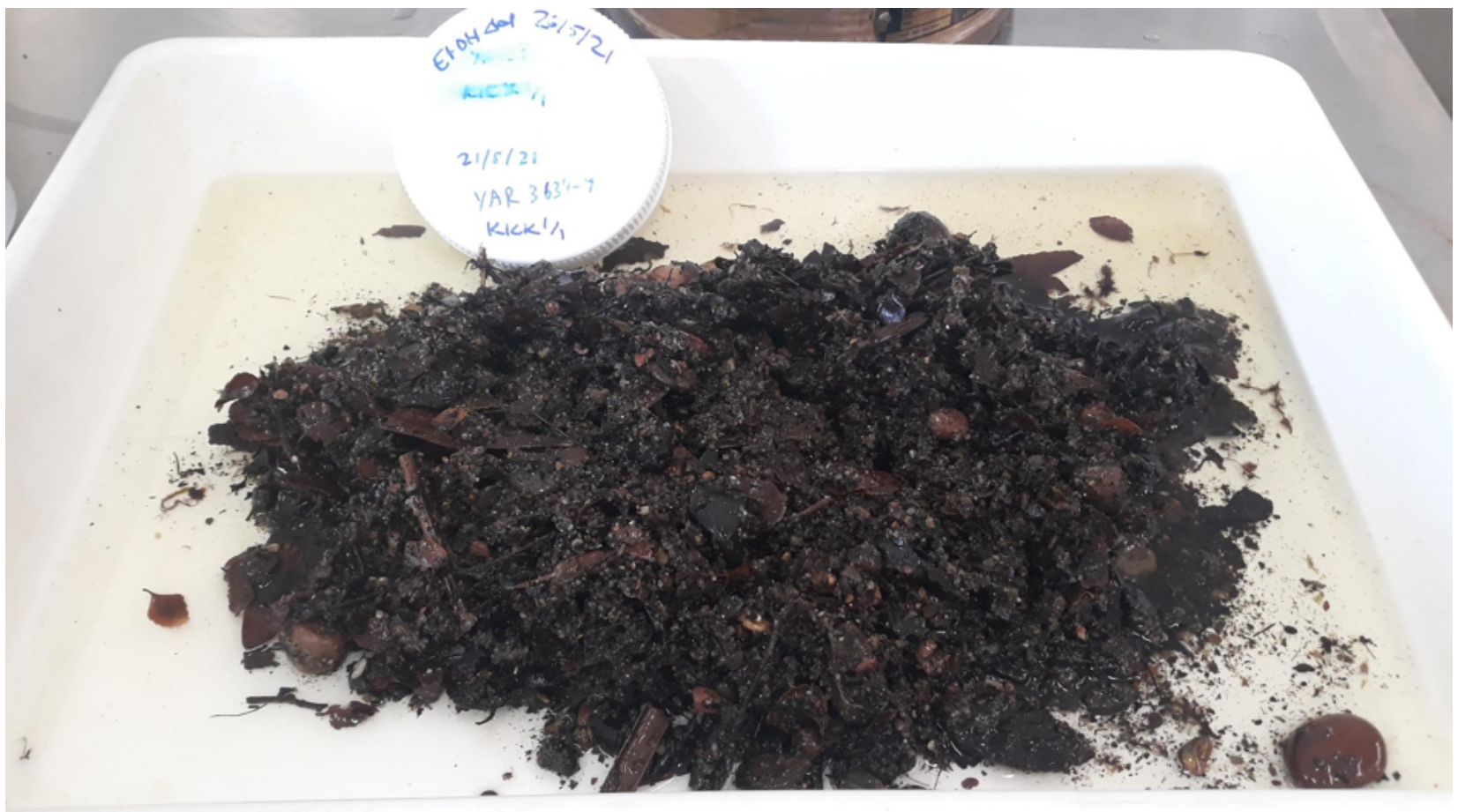

*Net-collected invertebrate sample*

- Place sieves from largest mesh size (at top) to smallest mesh size (at bottom). Place the stack of sieves in a large bucket (ideal the same diameter) into the sink and carefully decant the ethanol through sieves. (Keep window open or fumehood on for ethanol fumes)

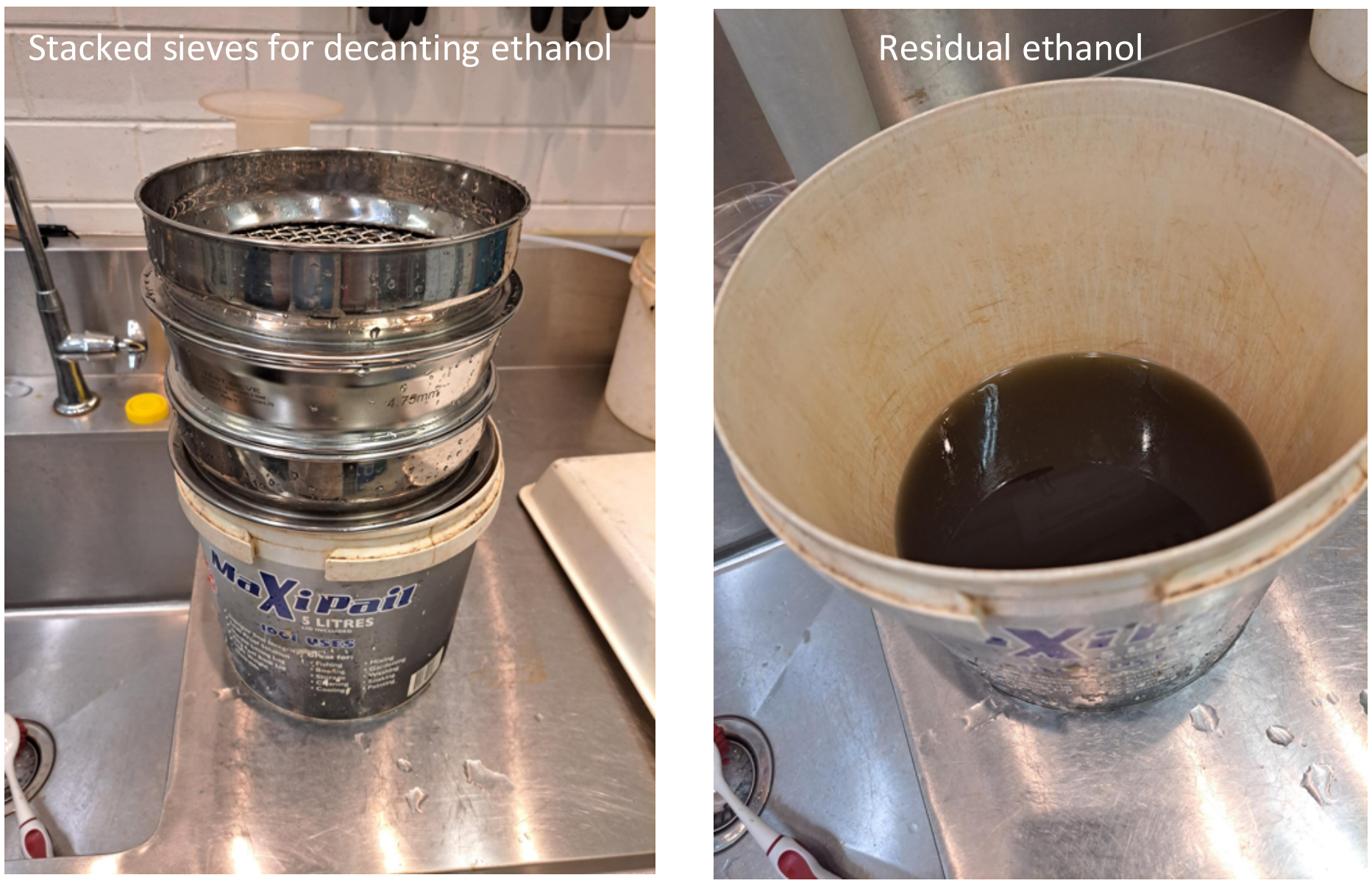


*Removing ethanol from samples prior to sieving*

- Retain bucket with ethanol and remove sieves as a stack and place in the sink on the extra sieve. The ethanol can be reused for storage of the retained sample fraction after sieving.

*Note: If sweep and kick samples are being combined for DNA metabarcoding they can be combined for sieving provided the volume of the samples is not too great to impede sieving.*

## STEP 2: Inspected sample -high sand content only

- Elutriate sand by flushing with high volumes of water to suspend the organic content and decant into the 250µm sieve (I find squeezing the end of the hose help float animals and other organics).
- Inspect remaining sand for macroinvertebrates. In some instances, samples will be elutriated in the field too. This will be indicated on the white sample container. The elutriated sand should be checked for animals.

- Place organic material retained from the elutriation step into a clean white sorting tray and place the sieve back in the sieve stack in sink. Proceed to Step 2

*Look out for on sand are usually snails, bivalves and caddisflies with stone cases*

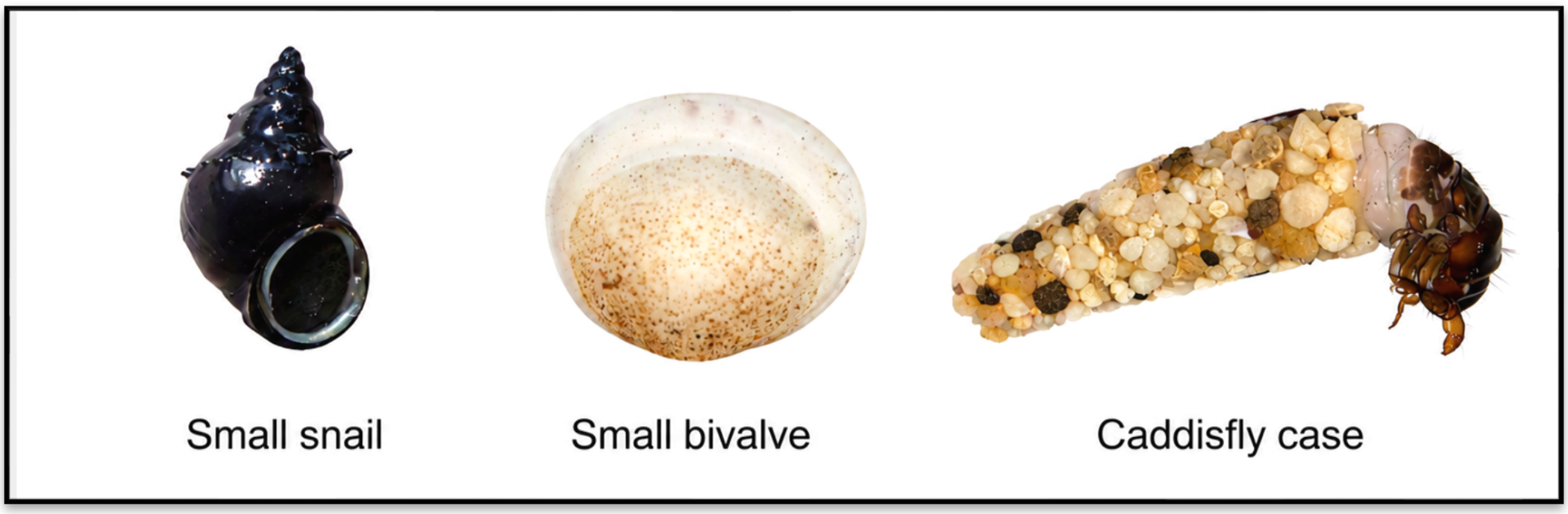


## STEP 3: Wash sample through sieves

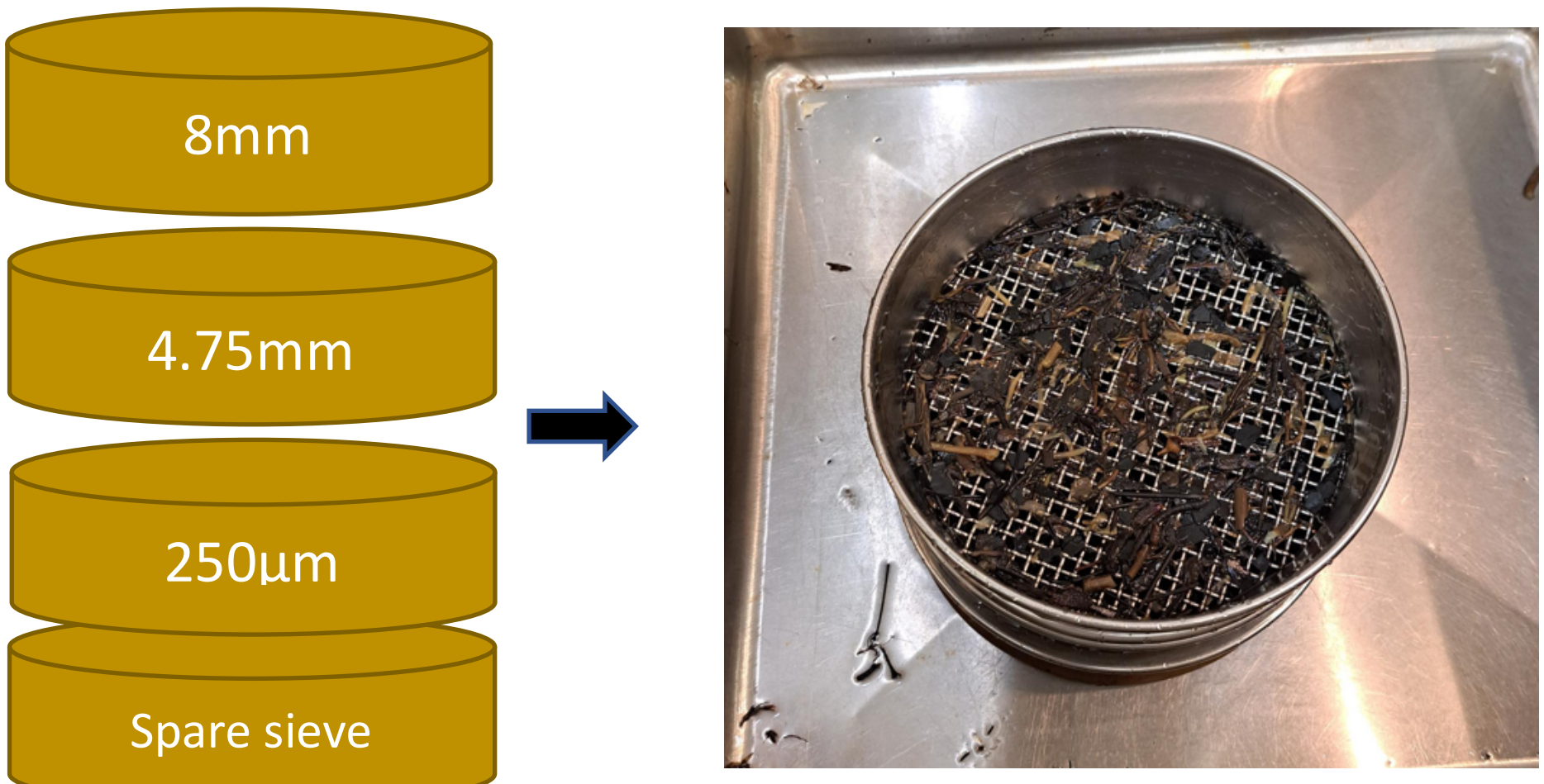


*Sieving sample through stacked sieves using water*

Wash any residual material from the sample containers into the white sorting tray. The sample container will be used to store the retained material.

- Wash the sample in portions through the stacked sieves. Large twigs, leaves, and stones can be removed from the sample (and placed in the spare sample container), but they must be washed thoroughly first. If the sample has a large volume or contains a lot of fine organic matter, the sieves may need to be emptied partway through sieving (see below), either into trays or by transferring the retained fraction (from the 250 µm sieve) into the sample container. *I tend to do this regularly while sieving.* The better a sample is washed, the less likely taxa will become trapped in the upper fractions, so tease apart algae or dense plant material and wash thoroughly.

***Note:*** *Look out for by-catch. Any obvious terrestrial species (e.g., earthworms, spiders, bees) or fish/tadpoles should be removed and not included in the retained fraction if encountered during sieving.*

## STEP 4 Search for large taxa in the >8 mm fraction and the 4.75–8 mm fraction

- Transfer all large taxa to the retained material. To date, only the very largest taxa—including **Crayfish, mussels,** and **shrimp** (when present in large numbers)—require subsampling. The presence of these taxa should be recorded in the sample-data Excel workbook, which will be checked prior to blending.

*Taxa to look out for in the 8mm sieve fraction*

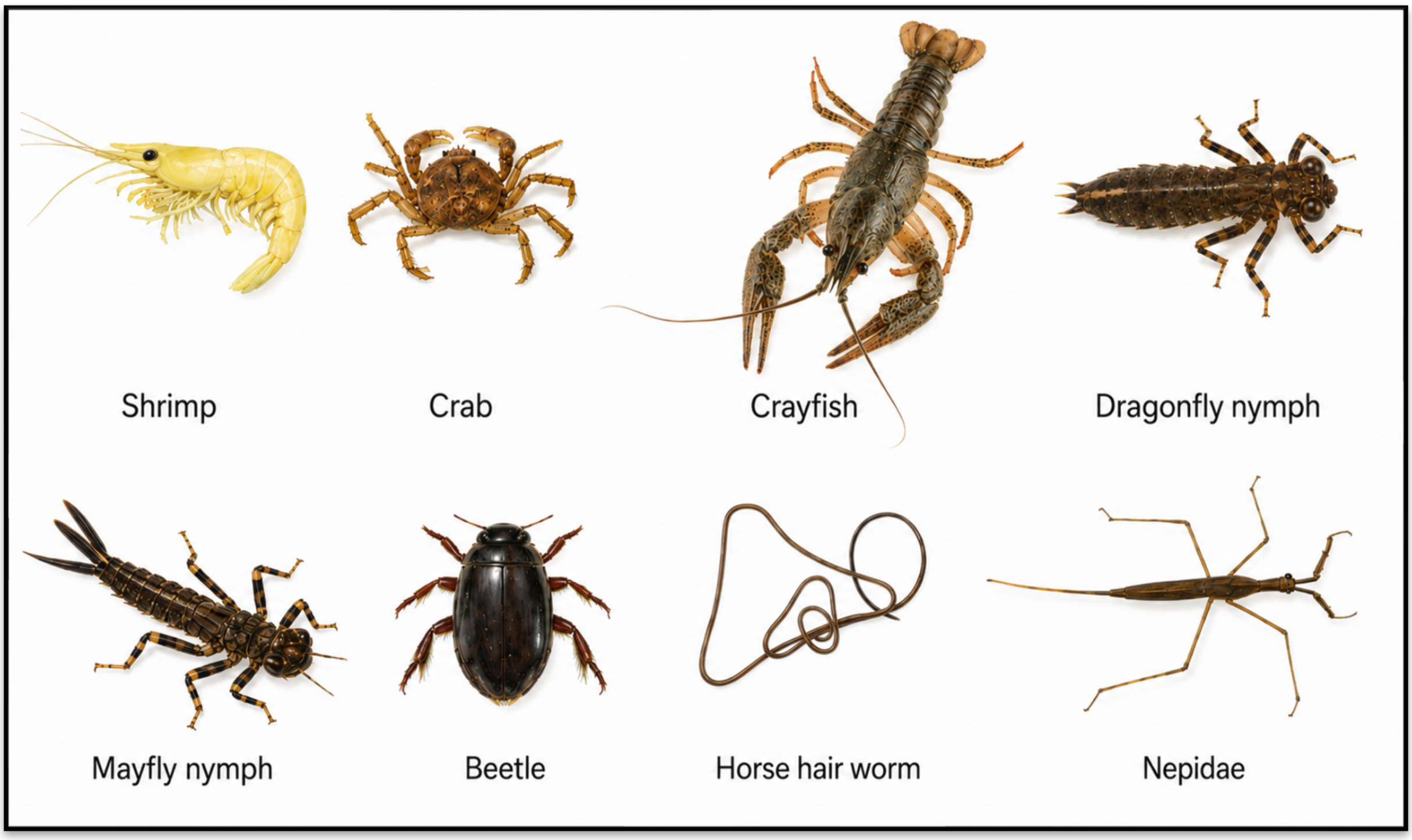


*Taxa to look out for in the 4.25 -8mm sieve fraction*

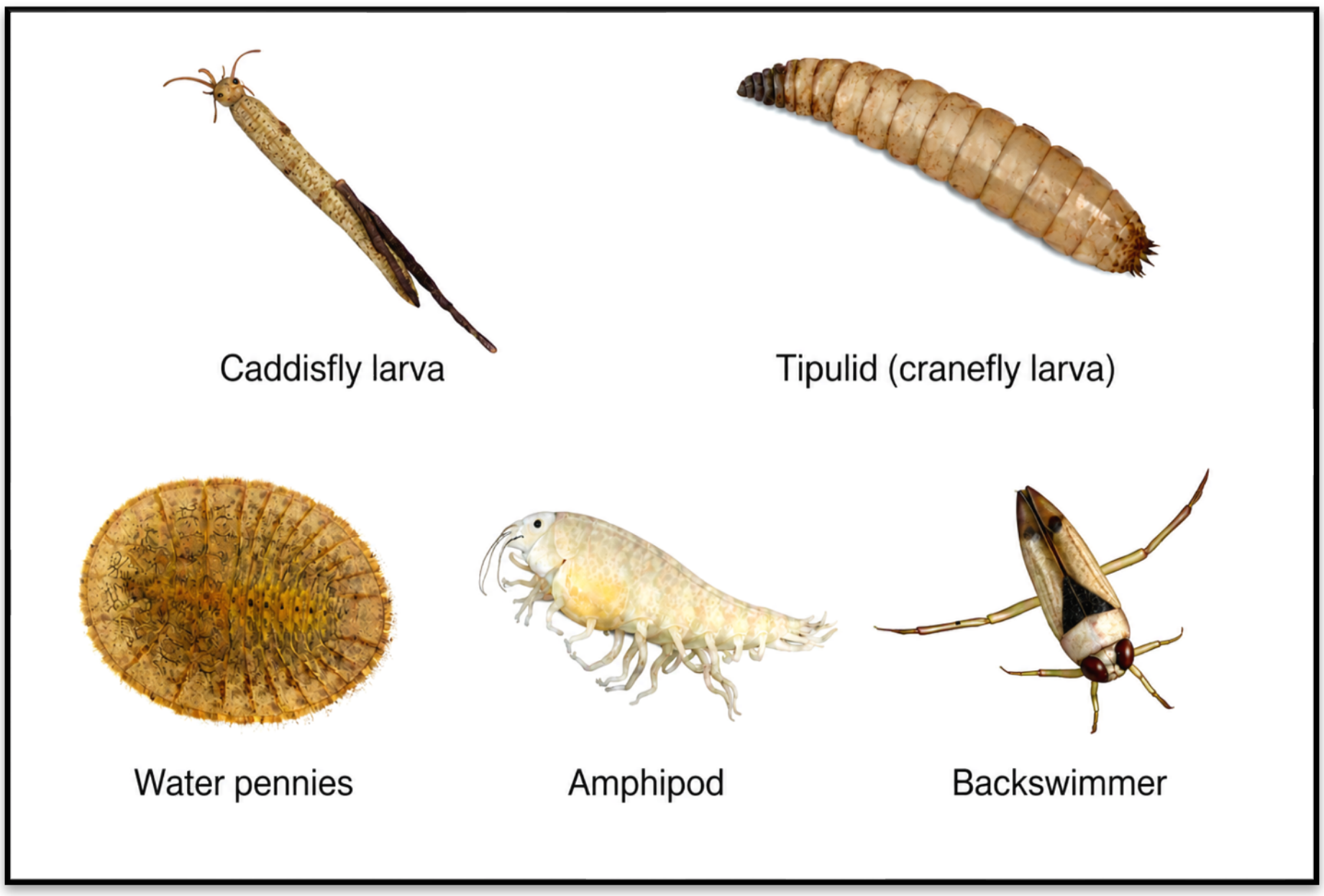


*Note: Most (97%) of the taxa picked from larger sieve fractions will also appear in the retained fraction (250µm - 4.75mm). Large, rare taxa should be favoured for being retained as these are less likely to be present in the retained fraction. It is important not to spend too much time picking small common taxa from the larger sieve fractions as this will increase the time spent on samples and will not necessarily improve species detection.*

## STEP 5: Keep and preserve the 250µm -4.75mm fraction

- Transfer the retained fraction into the retained sample container with all paper information labels. Label the lid with a permanent marker 'SIEVED' and the date so it does not get confused with sieved out material (can be placed in a separate container and have a large cross drawn on the lid).
- Record the sieving, date and any comments needed for blending (there are mussels in the sample, a large yabby, more than one sample container etc) on the sample data Excel workbook.
- The original ethanol decanted from the sample can be used to preserve the SIEVED sample until blending. The remaining content from the other fractions should not be required but can be kept short-term.
- Store both the retained fraction and the sieved-out material in the fridge.

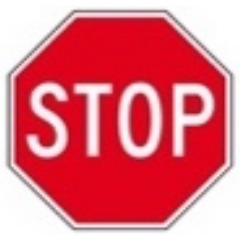


The trays, buckets and sieves should be washed thoroughly with hot soapy water and rinsed. They should then be inspected for any debris and animals before starting to process the next sample.
**Sieving should be completed in batches before proceeding to 'Step 6: Blending"**

## STEP 6: Blending

This is completed for the SIEVED samples **only** which contain macroinvertebrates for DNA metabarcoding.

- Check the sample data Excel workbook for any important notes prior to blending e.g., there are mussels in the sample, a large yabby, more than one sample container etc). For large animals (i.e., **yabbies, *Paratya*, freshwater mussels**) only a portion of the animal is added. The remainder of these animals can be stored in labelled vials.
- Transfer the SIEVED sample to the blender (remove paper labels first). Blend for a 1-2 mins stopping the blender at least once (to dislodge material from the lid).

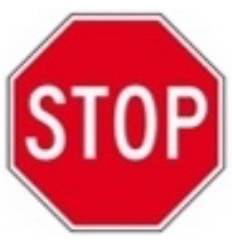


The glass blender container should be thoroughly washed in warm soapy water, then rinsed and dried between samples.
**Completing the blending in batches is the most efficient way to process samples. Wear ear protection.**

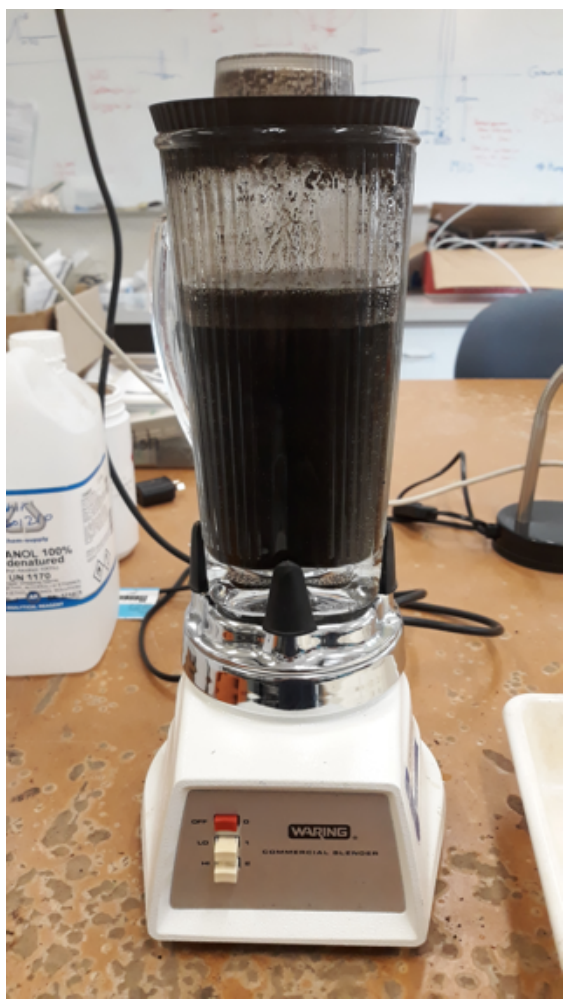

*Sample after blending*

## STEP 7: Drying

- After blending, samples need to sit (hours or overnight), so the organic material settles and can mostly separate from ethanol. The excess ethanol should be decanted into a bucket (and disposed of as ethanol waste).
- The sample can be placed in a fast-food container for drying, with paper labels and labelled with a permanent marker on the outside of both ends of the container (also indicate how many trays there are, often more than one is needed)

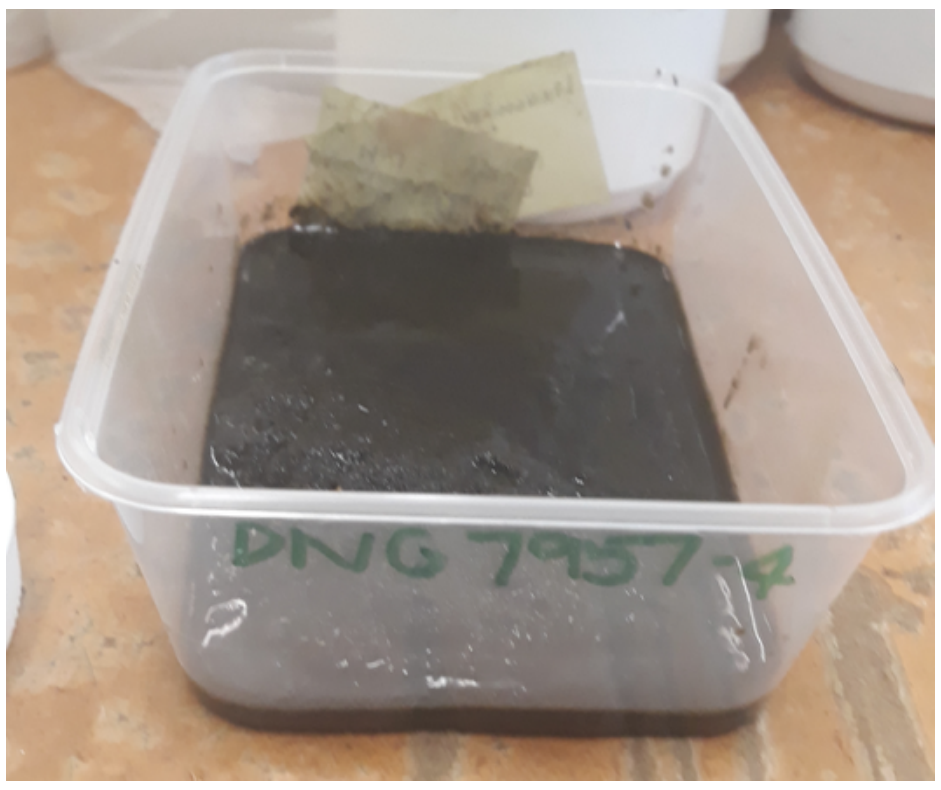


*Sample ready for drying*

- Open containers should be labelled with the sample information and carefully placed in a convection oven at 55°C overnight to evaporate off residual ethanol (Note some samples may need longer than 24 hrs). Start at the top of the oven. When samples are dry place lids on and put in the -20°C freezer.

*Note: Turn on fans when drying to stop a build-up of ethanol fumes*

## STEP 8: Weighing

- Place an empty fast-food container on the balance and zero the balance. The dried samples can now be weighed for the total dry weight. This can be repeated for all dried and blended samples. Record dry weight in the sample data Excel workbook.
- Calculate 30% of the dry weight (this can be set up in sample data Excel workbook) and weigh out the amount on the lid (re-zero the balance first).
- Transfer dry material to a 50 mL falcon tube. Ensure the volume in the tube DOES NOT exceed 40 mL. Use extra tubes if required (but this needs to be indicated on labels and recorded in sample data Excel workbook). Ensure tubes are fully labelled with the sample name on the lid and side of the tube using a permanent marker. Clean between samples.
-

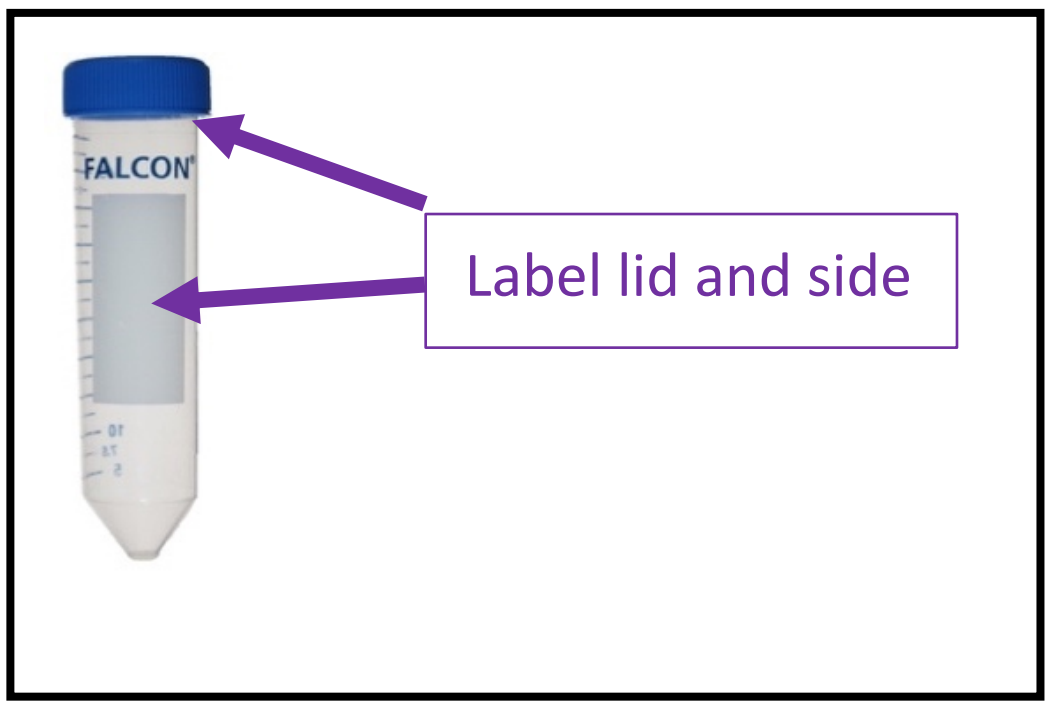


Samples can be stored in the -80°C freezer (at UoM Parkville) to await DNA extraction. Please proceed to the next manual.
