# Supplementary material for: Simplifying Macroinvertebrate Biodiversity Assessment: A Standardised Metabarcoding Method for Invertebrate Samples With High Volumes of Debris
Source: Mol Ecol Resour. 2026 Oct 4;26(7):e70213. doi: 10.1111/1755-0998.70213 (PMC13635761; doi:10.1111/1755-0998.70213)
Supplement: Supplementary file 5 [file MEN-26-e70213-s006.pdf]

00_preparing files for preprocessing.Rmd
- creates a mapping file from demultiplexed Miseq fastq.gz for QIIME2

01_preprocessing.Rmd:
- Load python environment, functions and raw MiSeq data files
- Load Primer/Amplicon information
  - The ‘ref’ folder can be configured with any primer combinations
- **Demux** (splits raw data by sample and removes adaptors)
- **Split** (splits samples by amplicon)
- **Trim** (removes forward and reverse primers)
- **Summary** (talles read counts for per amplicon)
- **QC plots**
  - Used to guide selection of amplicon truncation lengths for DADA2

02_quantification.Rmd:
- **Combine** (For each amplicon combine all data for DADA2)
- **DADA2** (quality filtering, error model learning, denoising, dereplication, Paired-end merging, chimera removal, ASV table construction)
- Low frequency ASV filtering (if required)
- **LULU** (additional ASV denoising - combines similar ASVs**)**

03_classification.Rmd:
- Load **QIIME2** reference libraries (Custom amplicon-specific reference libraries need to be compiled from public (GenBank, BOLD systems) and in-house databases)
- **VSEARCH** searches ASVs from each amplicon against amplicon-specific reference libraries

04_final_data_summary.Rmd:
- Brings together all bioinformatic data into a single data frame
  - AVS read counts, classification and sequences for each amplicon

05_rarefaction.Rmd:
- Rarefaction plots for each amplicon to assess sequencing depth

06_filling_taxonomy_PCR_rep_filtering.Rmd
- Fills taxonomy using a vetted library of ASVs
- Filters data to remove tag switches and low-level contamination
  - filtering parameters can be set using control samples as a guide

07_data_analysis.Rmd
- R scripts for visualizing run statistics and analyzing data

Packages: R packages | Cutadapt (QIIME2) | QIIME2 | DADA2 (QIIME2) | LULU | VSEARCH (QIIME2)
